# Computational Analysis of Some More Rectangular Tessellations of Kekulenes and Their Molecular Characterizations

**DOI:** 10.3390/molecules28186625

**Published:** 2023-09-14

**Authors:** S. Prabhu, M. Arulperumjothi, Muhammad Usman Ghani, Muhammad Imran, S. Salu, Bibin K. Jose

**Affiliations:** 1 Department of Mathematics, Rajalakshmi Engineering College, Chennai 602105, India; 2 Department of Mathematics, St. Joseph’s College of Engineering, Chennai 600119, India; marulperumjothi@gmail.com; 3 Institute of Mathematics, Khawaja Fareed University of Engineering & Information Technology, Abu Dhabi Road, Rahim Yar Khan 64200, Pakistan; usmanghani85a@gmail.com; 4 Department of Mathematical Sciences, United Arab Emirates University, Al Ain P.O. Box 15551, United Arab Emirates; 5 PG & Research Department of Mathematics, Sanatana Dharma College, Kerala University, Kerala 688003, India; salusnath@gmail.com (S.S.); bibinkjose2002@gmail.com (B.K.J.)

**Keywords:** distance, molecular descriptors, convex subgraph, molecular modeling of kekulene

## Abstract

Cycloarene molecules are benzene-ring-based polycyclic aromatic hydrocarbons that have been fused in a circular manner and are surrounded by carbon–hydrogen bonds that point inward. Due to their magnetic, geometric, and electronic characteristics and superaromaticity, these polycyclic aromatics have received attention in a number of studies. The kekulene molecule is a cyclically organized benzene ring in the shape of a doughnut and is the very first example of such a conjugated macrocyclic compound. Due to its structural characteristics and molecular characterizations, it serves as a great model for theoretical research involving the investigation of π electron conjugation circuits. Therefore, in order to unravel their novel electrical and molecular characteristics and foresee potential applications, the characterization of such components is crucial. In our current research, we describe two unique series of enormous polycyclic molecules made from the extensively studied base kekulene molecule, utilizing the essential graph-theoretical tools to identify their structural characterization via topological quantities. Rectangular kekulene Type-I and rectangular kekulene Type-II structures were obtained from base kekulene molecules arranged in a rectangular fashion. We also employ two subcases for each Type and, for all of these, we derived ten topological indices. We can investigate the physiochemical characteristics of rectangular kekulenes using these topological indices.

## 1. Introduction

Organic compounds known as polycyclic aromatic hydrocarbons are composed of two or more single or fused aromatic rings with a pair of carbon atoms shared between rings in their molecules [1,2,3,4]. Having various structures and toxicities, they are environmentally stable compounds with low vapor pressure, aqueous solubility, and high boiling points and melting points [5,6]. These polycyclic aromatics’ chemical and bioactive properties have generated a great deal of interest in a variety of industries, including liquid crystals, medicines, agriculture, electronics, functional plastics, and photographic products [7]. They are also classified as pollutants of the environment because of their toxic and carcinogenic nature; thus, studying how they affect the ecosystem enables us to develop corrective actions for both biological and physical systems [7,8]. Due to their improved stabilization caused by the macrocyclic conjugation, these compounds also exhibit the superaromaticity phenomenon, which has recently attracted the interest of theoretical and experimental investigation [4,5,7,9].

In terms of the issue of π-bond delocalization, cycloarenes are a fascinating class of aromatic systems. They are produced by combining linear and angular annelations of benzene units, and they contain fully-annealed macrocyclic structures that enclose avoid that carbon–hydrogen bonds point into [10,11,12,13,14]. McWeeny was the one who first proposed their electronic structure [15,16], though several attempts had been undertaken to synthesize them since 1951. Nearly 30 years later, the pioneer in this sector, cyclo[d.e.d.e.d.e.d.e.d.e.d.e] dodecakisbenzene—C_48_H_24_, as a chemical formula—was effectively synthesized [17]; it later came to be known as kekulene. At a later time, Kumar et al. [18] and Funhoff et al. [19] synthesized two further novel cycloarene compounds, septulene and cyclo[d.e.d.e.e.d.e.d.e.e] decakisbenzene. Surprisingly, there are few Clar structures with such fragrant sextets, in sharp contrast to the highly conjugated character of cycloarenes [20,21]. Over the years, there has been a lot of interest in the synthesis and characterization of these intriguing molecules because of their paradoxical behavior and the possibility that the entire molecule contains globally delocalized electrons [10,11,12,13,14,22,23].

With twelve annelated benzene rings making up a complete cycle, the structure of kekulene presents the enticing promise of improved stabilization with intriguing magnetic characteristics and magnetocaloric effects. Using ab initio methods, it was discovered that the current density maps display global diamagnetic and parameter circulations along the inner and outer perimeters that include six local benzenoid diamagnetic π circulations [24]. It was revealed through a comparison analysis of this compound’s geometric and magnetic properties with those of phenanthrene; anthracene; and 1, 2:7, 8-dibenzanthracene that it has six benzenoid sextets and a D_6*h*_ Clar structure, making it a typical aromatic molecule [25]. Although this molecule has remarkable physicochemical characteristics, its uses have long been restricted due to its difficult synthesis procedures [11]. Pozo et al. [26] recently addressed this issue and established an effective approach for the synthesis of kekulene using aryne chemistry. As a result, it makes it possible for large-scale manufacture, which, in turn, draws chemists’ attention to the investigation of its exciting applications. It was then suggested that this structure’s transition order from ferromagnetic (FM) to paramagnetic (PM) is a second-order process, making it an eligible candidate for magnetic refrigeration [27]. In addition, the compound’s unique mix of qualities including strong Li mobility, high cell voltage, and extremely high storage capacity enables it to be a practical material for anodes for lithium-ion batteries [28]. This unique structure’s theoretical characterization will, therefore, be important for further research into its intriguing characteristics and potential uses.

From this point forward, Graph theory, group theory, and combinatorial mathematics’ enumerative mathematical tools are needed to improve the exhaustive production and synthesis of these prospective novel compounds. These theoretical techniques mainly rely on molecular similarity measurements and associated tenets; they are essential for assisting computer-aided drug discovery (CADD) procedures. Any parameter that explores the topology of the chemical structure is found to be extremely useful in the characterization of novel biological structures and DNA permutations due to the intimate relationship between the pharmacological and biomedical attributes and their subatomic configurations that is revealed by a large number of medication tests. One class of molecular parameters known as topological indices characterizes the structural geometry of molecules to describe the size and shape of chemical species [29,30,31]. They offer crucial information for understanding a variety of physicochemical characteristics, such as chromatographic retention times, anesthetic potency, carcinogenic behavior, and aromaticity [29,32,33,34]. With the aid of these descriptors, the theoretical possibility of examining and manipulating a newly synthesized compound is increased, which, in turn, enables us to select between molecular structures that deserve to be synthesized and tested and those that are not, allowing us to significantly lower the cost of our experimental work. The importance of mathematical and computational techniques in helping pharmaceutical and medical professionals understand the biological and chemical properties of novel medications was well demonstrated by a recent review [35,36].

By converting the compound’s atomic structure into a molecular graph, where every vertex denotes a single atom and every edge represents a chemical bond across atoms, the topological descriptors of the compound are numerically established. It is necessary to assign each compound with a specific numerical code in order to study the relationships between the actual chemical structure and its biological and physicochemical activities; further, enabling the investigation of correlations between quantitative structure–activity relationships (QSAR), quantitative structure–toxicity relationships (QSTR), quantitative structure–property relationships (QSPR), and topological indices makes this encoding possible [37,38,39]. These topological descriptors’ ciphers provide the necessities for successful examination of biological and medical information of new medications without the need for experimental apparatus by encoding a quantitative measure for each molecule based on its underlying topology, which is often graph-invariant in nature. As a result, topological indices and their subsequent advances have received a lot of interest, and many papers are now devoted to the research of novel molecular descriptors and the associated computational techniques [29,40,41,42,43,44,45,46,47]. Some further works on topological indices and related topics can be seen in [48]; we can extend our future works to these areas [49,50,51,52], and numerous similar issues can be solved if new topological indices are defined or modified. Through the use of strength-weighted graphs, this study establishes the topological characterization of rectangular kekulene meshes Type I and II using a variety of Bond additive, distance-based, distance- and degree-based, associated topological descriptors, thus enhancing our understanding of the numerous chemical and physical characteristics of these compounds, such as their aromaticity, topological resonance energy, ring current diamagnetism, and the functions performed by graph-theoretical internal substructures like conjugated circuits.

## 2. Background

An ordered pair (V,E) is a graph in which *V* stands for the set of vertices and *E* for the set of edges. The degree of a vertex *v* is a non-negative number denoted as $d_{G}\left(v\right)$ that indicates how many edges have entered the vertex. How many edges there are in a shortest path determines the distance between two vertices *u* and *v* in a graph *G*, which is indicated by the symbol $d_{G}\left(u,v\right)$. Thus, an edge f=xy and a vertex *u* with distance $d_{G}\left(u,f\right)$ is defined as the minimum of $\left\{d_{G}\left(u,y\right),d_{G}\left(u,x\right)\right\}$; meanwhile, the minimum of $\left\{d_{G}\left(v,f\right),d_{G}\left(u,f\right)\right\}$ with the distance between two edges e=uv and f=xy is denoted by $D_{G}\left(e,f\right)$. The collection of vertices that are adjacent to a vertex *u* is said to be its neighborhood, or $N_{G}\left(u\right)$. If $d_{G}\left(u,v\right)=d_{H}\left(u,v\right)$ for a subgraph *H* of *G*, then *H* is known as *G*’s isometric subgraph, and the convex subgraph *H* of *G* is known if the whole shortest path in *H* connecting any pair of vertices lies entirely within *H*. We denote $N_{u}\left(e\right|G)$ and $M_{u}\left(e\right|G)$ to the collection of *G*’s vertices and edges, respectively, which are nearer to *u* than *v* for an edge e=uv∈E(G) and $n_{u}\left(e\right|G)$, and $m_{u}\left(e\right|G)$ represent how many members there are in these collections. Similarly, $n_{v}\left(e\right|G)$ and $m_{v}\left(e\right|G)$ are defined. Denote {1,2,…,η} by [η] and {η+1,η+2,…,2η+1} by [2η+1]−[η]. Also, we denote the strength-weighted quotient graph by SWQG and the quotient graph by QG.

The first appearance of the strength-weighted graph (SWG) was in [45] and extensively addressed in [46,47,53,54,55,56,57,58,59,60] as $G_{sw}=\left(G,\left(w_{v},s_{v}\right),s_{e}\right)$, where $w_{v}:V\left(G_{sw}\right)\to R_{0}^{+}$ is the vertex-weight, $s_{v}:V\left(G_{sw}\right)\to R_{0}^{+}$ is the vertex-strength, and $s_{e}:E\left(G_{sw}\right)\to R_{0}^{+}$ is the edge-strength. In the strength-weighted graph, $d_{G_{sw}}\left(u,v\right)=d_{G}\left(u,v\right)$, $d_{G_{sw}}\left(u,f\right)=d_{G}\left(u,f\right)$, $D_{G_{sw}}\left(e,f\right)=D_{G}\left(e,f\right)$, $N_{u}\left(e\right|G_{sw})=N_{u}\left(e\right|G)$, and $M_{u}\left(e\right|G_{sw})=M_{u}\left(e\right|G)$. In order to determine the measures of closeness cardinality, $n_{u}\left(e\right|G_{sw})=\sum_{x\in N_{u}\left(e\right|G_{sw})}w_{v}\left(x\right),$ $m_{u}\left(e\right|G_{sw})=\sum_{x\in N_{u}\left(e\right|G_{sw})}s_{v}\left(x\right)+\sum_{f\in M_{u}\left(e\right|G_{sw})}s_{e}\left(f\right)$, and $t_{u}\left(e\right|G_{sw})=n_{u}\left(e\right|G_{sw})+m_{u}\left(e\right|G_{sw})$. The computations of $n_{v}\left(e\right|G_{sw})$, $m_{v}\left(e\right|G_{sw})$ and $t_{v}\left(e\right|G_{sw})$ are similar. A vertex degree in $G_{sw}$ of *u* is determined by $d_{G_{sw}}\left(u\right)=\sum_{x\in N_{G_{sw}}\left(u\right)}s_{e}\left(ux\right)$. The critically vital distance-based topological indices (TI) of $G_{sw}$ are now being shown and take notice of the equality of $TI(G_{sw})$ and TI(G) when $s_{e}=1$, $w_{v}=1$, and $s_{v}=0$.

1. Wiener $W\left(G_{sw}\right)=\sum_{\left\{u,v\right\}\subseteq V\left(G_{sw}\right)}w_{v}\left(u\right)w_{v}\left(v\right)d_{G_{sw}}\left(u,v\right)$;
2. Edge-Wiener $W_{e}\left(G_{sw}\right)=\sum_{\left\{u,v\right\}\subseteq V\left(G_{sw}\right)}s_{v}\left(u\right)\;s_{v}\left(v\right)\;d_{G_{sw}}\left(u,v\right)+\sum_{\left\{e,f\right\}\subseteq E\left(G_{sw}\right)}s_{e}\left(e\right)\;s_{e}\left(f\right)\;D_{G_{sw}}\left(e,f\right)$
  $\;+\;\sum_{u\in V(G_{sw})}\sum_{f\in E(G_{sw})}s_{v}\left(u\right)\;s_{e}\left(f\right)\;d_{G_{sw}}\left(u,f\right)$;
3. Vertex-edge-Wiener $W_{ve}\left(G_{sw}\right)=\frac{1}{2}[\sum_{\left\{u,v\right\}\subseteq V\left(G_{sw}\right)}\left\{w_{v}\left(u\right)s_{v}\left(v\right)+w_{v}\left(v\right)s_{v}\left(u\right)\right\}\;d_{G_{sw}}\left(u,v\right)\;$
  $+\sum_{u\in V(G_{sw})}\sum_{f\in E(G_{sw})}w_{v}\left(u\right)\;s_{e}\left(f\right)\;d_{G_{sw}}\left(u,f\right)]$;
4. Vertex-Szeged $Sz_{v}\left(G_{sw}\right)\;=\sum_{e=uv\in E(G_{sw})}s_{e}\left(e\right)n_{u}\left(e\right|G_{sw})n_{v}\left(e\right|G_{sw})$;
5. Edge-Szeged $Sz_{e}\left(G_{sw}\right)\;=\sum_{e=uv\in E(G_{sw})}s_{e}\left(e\right)m_{u}\left(e\right|G_{sw})m_{v}\left(e\right|G_{sw})$;
6. Edge-vertex-Szeged $Sz_{ev}\left(G_{sw}\right)=\frac{1}{2}\sum_{e=uv\in E(G_{sw})}s_{e}\left(e\right)[n_{u}\left(e\right|G_{sw})m_{v}\left(e\right|G_{sw})+$ $n_{v}\left(e\right|G_{sw})m_{u}\left(e\right|G_{sw})]$;
7. Total-Szeged $Sz_{t}\left(G_{sw}\right)\;=Sz_{v}\left(G_{sw}\right)+Sz_{e}\left(G_{sw}\right)+2Sz_{ev}\left(G_{sw}\right)$;
8. Padmakar–Ivan $PI\left(G_{sw}\right)\;=\sum_{e=uv\in E(G_{sw})}s_{e}\left(e\right)\left[m_{u}\left(e\right|G_{sw})+m_{v}\left(e\right|G_{sw})\right]$;
9. Schultz $S\left(G_{sw}\right)\;=\sum_{\left\{u,v\right\}\subseteq V\left(G_{sw}\right)}\left[w_{v}\left(v\right)\left(d_{G_{sw}}\left(u\right)+s_{v}\left(u\right)\right)+w_{v}\left(u\right)\left(d_{G_{sw}}\left(v\right)+s_{v}\left(v\right)\right)\right]d_{G_{sw}}\left(u,v\right)$;
10. Gutman $Gut\left(G_{sw}\right)\;=\sum_{\left\{u,v\right\}\subseteq V\left(G_{sw}\right)}\left[\left(d_{G_{sw}}\left(u\right)+s_{v}\left(u\right)\right)\left(d_{G_{sw}}\left(v\right)+s_{v}\left(v\right)\right)\right]d_{G_{sw}}\left(u,v\right)$.

For handling topological descriptors based on distance, the cut method approach is incredibly beneficial [61,62]. It was effectively used very frequently to compute distance-based topological indices for benzenoid frameworks [45,46,55,56,63]. The Djoković–Winkler Θ relation is the fundamental concept behind the cut method, which has the following definition: Regarding any pair of edges of *G* with e=uv and f=cd, $d_{G}\left(u,c\right)+d_{G}\left(v,d\right)\neq d_{G}\left(u,d\right)+d_{G}\left(v,c\right)$, the relation Θ is symmetric and reflexive, though not necessarily transitive. However, its transitive closure $\Theta^{*}$ results in the formation of an equivalence relation. Let $F=\{F_{1},F_{2},\ldots F_{r}\}$ represent the *G*’s $\Theta^{*}$-partition and every class $F_{i}$; the disconnected graph $G-F_{i}$ is utilized to generate the quotient graph $G/F_{i}$, in which vertices of $G/F_{i}$ are connected components of $G-F_{i}$ and $C_{j}^{i}$, and $C_{k}^{i}$ are two adjacent components whenever vertex $x\in C_{j}^{i}$ and vertex $y\in C_{k}^{i}$ are adjacent to one another such that $xy\in F_{i}$. It is argued that a partition $E=\{E_{1},E_{2},\ldots E_{k}\}$ of E(G) is coarser than F of E(G) if every set $E_{i}$ is expressed as the union of at least one $\Theta^{*}$-class of *G*. For details on TIs with respect to this notation, see [40,45,46].

## 3. Rectangular Kekulene Systems

In light of its significance in numerous scientific domains, including electronics [64], organic photovoltaics [65], and optoelectronic devices [66], polycyclic aromatic hydrocarbons have become the subject of extensive experimental and theoretical research. Some of the larger polycyclic compounds are often used as a cutout and model for materials made of glassy carbon and graphite sheets [67]. In general, these large polycyclics are totally made of condensed hexagon rings either by increasing the basic molecule’s size; by circumscribing it in benzene rings; or by a basic molecule that is squeezed into oligomers, trimers, and dimers [68,69,70]. A number of TIs for a 2D sheet made up of numerous kekulenes were computed in [53]. In this study, we thoroughly examine two of these new hollow-sited PAH compounds made from kekulene structures.

**Figure 1:**
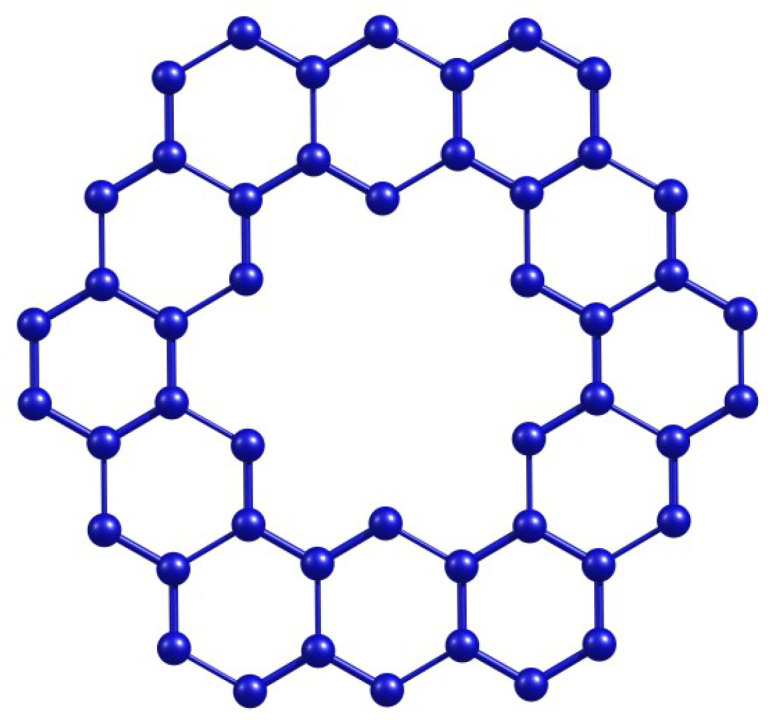
Kekulene molecular structure.

**Figure 2:**
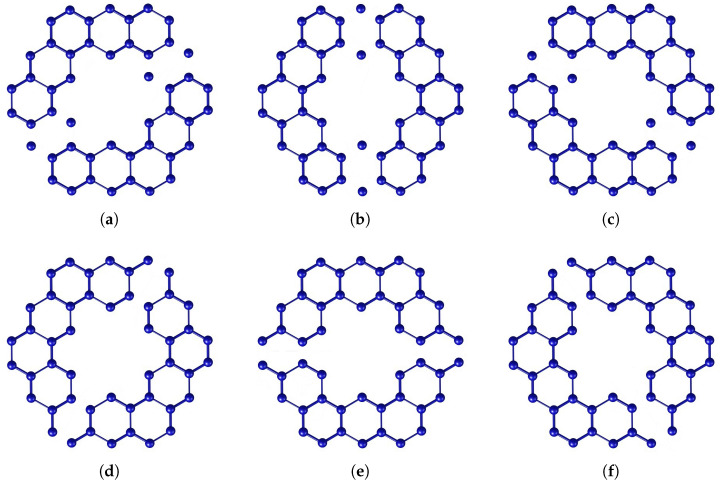
(**a**) Zigzag Acute. (**b**) Zigzag Vertical. (**c**) Zigzag Obtuse. (**d**) Acute. (**e**) Horizontal. (**f**) Obtuse.

Figure 1 shows the kekulene molecule’s basic chemical structure. Additionally, it is used in different configurations to create new series of big PAH compounds that are based on kekulenes. The different $\Theta^{*}$-classes of kekulene structure that are crucial to our computation in large polycyclic aromatic compounds are now illustrated. The $\Theta^{*}$-classes are zigzag vertical (VRZ), zigzag acute (ACZ), zigzag obtuse (OBZ), horizontal (TH,H), obtuse (OB), and acute (AC), as shown in Figure 2.

### Kekulene

**Figure 3:**
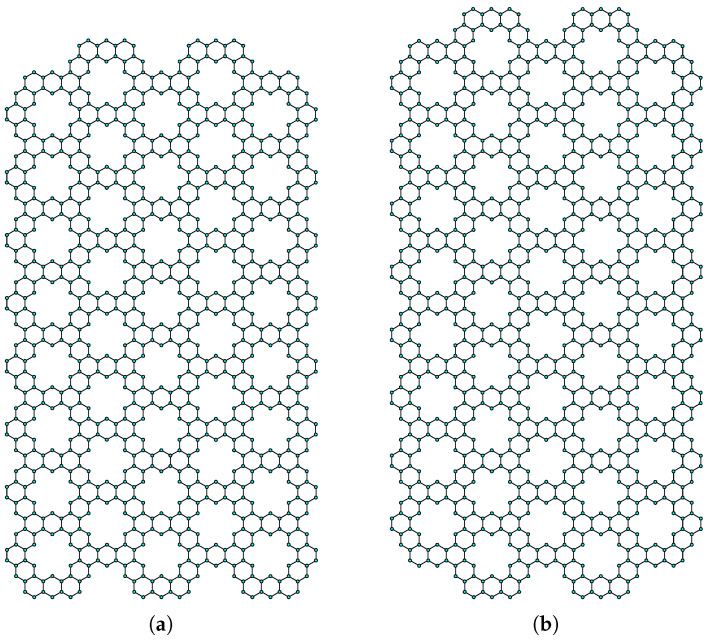
(**a**) Kekulene Type-I. (**b**) Kekulene Type-II.

The kekulene units are arranged in a rectangular mesh configuration, as depicted in Figure 3a, to form the rectangular kekulene system RK(m,n) Type-I. It has 36mn−2m+32n−18 number of vertices and 48mn+40n−4m−24 number of edges. The kekulene units are arranged in a rectangular mesh configuration, as depicted in Figure 3b, to form a RK(m,n) Type-II rectangular kekulene system. It has 36mn+50n−2m−36 number of vertices and 48mn+64n−4m−48 number of edges. Now, for this planar tiling-based kekulene system, we calculate the distance-based topological indices.

**Theorem** **1.** *Let G be a RK(m,n) Type-I rectangular kekulene system with m=3n−1.*

(i) *Wiener*
  - *$W\left(G\right)=\left(19440m^{3}n^{2}-2160m^{3}n+60m^{3}+12960m^{2}n^{3}+49680m^{2}n^{2}-34080m^{2}n+1560m^{2}+123120mn^{4}-1080mn^{3}-2250mn^{2}-29550mn+765m-106272n^{5}+134100n^{4}-42680n^{3}-5880n^{2}+5372n-3015\right)/15$.*
(ii) *Edge-Wiener*
  -
$W_{e}\left(G\right)=\left(34560m^{3}n^{2}-5760m^{3}n+240m^{3}+23040m^{2}n^{3}+69120m^{2}n^{2}-59160m^{2}n+4020m^{2}+218880mn^{4}-43200mn^{3}-27960mn^{2}-24180mn+210m-188928n^{5}+248160n^{4}-109120n^{3}-3360n^{2}+26488n-11310\right)/15.$

(iii) *Vertex-Edge-Wiener*
  -
$W_{ve}\left(G\right)=\left(34560m^{3}n^{2}-5760m^{3}n+240m^{3}+23040m^{2}n^{3}+69120m^{2}n^{2}-59160m^{2}n+4020m^{2}+218880mn^{4}-43200mn^{3}-27960mn^{2}-24180mn+210m-188928n^{5}+248160n^{4}-109120n^{3}-3360n^{2}+26488n-11310\right)/15.$

(iv) *Vertex-Szeged*
  -
$Sz_{v}\left(G\right)=\left(103680m^{3}n^{3}-12960m^{3}n^{2}-4080m^{3}n+279360m^{2}n^{3}-175200m^{2}n^{2}+14160m^{2}n-1080m^{2}+699840mn^{5}-146880mn^{4}+59000mn^{3}-202800mn^{2}+64300mn-7380m-699840n^{6}+784224n^{5}-159160n^{4}-6000n^{3}-66560n^{2}+28476n-4620\right)/15.$

(v) *Edge-Szeged*
  -
$Sz_{e}\left(G\right)=\left(184320m^{3}n^{3}-40320m^{3}n^{2}-960m^{3}n-480m^{3}+433920m^{2}n^{3}-339840m^{2}n^{2}+50160m^{2}n-5160m^{2}+1244160mn^{5}-453120mn^{4}+57760mn^{3}-333600mn^{2}+153320mn-20100m-1244160n^{6}+1462272n^{5}-437760n^{4}+29280n^{3}-104400n^{2}+76608n-21540\right)/15.$

(vi) *Edge-Vertex-Szeged*
  -
$Sz_{ev}\left(G\right)=\left(138240m^{3}n^{3}-23760m^{3}n^{2}-3240m^{3}n-120m^{3}+348960m^{2}n^{3}-245040m^{2}n^{2}+28080m^{2}n-2520m^{2}+933120mn^{5}-267840mn^{4}+57840mn^{3}-260760mn^{2}+100440mn-12180m-933120n^{6}+1071168n^{5}-274120n^{4}+6320n^{3}-83120n^{2}+47932n-11040\right)/15.$

(vii) *Total-Szeged*
  -
$Sz_{t}\left(G\right)=\left(564480m^{3}n^{3}-100800m^{3}n^{2}-11520m^{3}n-720m^{3}+1411200m^{2}n^{3}-1005120m^{2}n^{2}+120480m^{2}n-11280m^{2}+3810240mn^{5}-1135680mn^{4}+232440mn^{3}-1057920mn^{2}+418500mn-51840m-3810240n^{6}+4388832n^{5}-1145160n^{4}+35920n^{3}-337200n^{2}+200948n-48240\right)/15.$

(viii) *Padmakar–Ivan*
  -
$PI\left(G\right)=\left(4608m^{2}n^{2}-1008m^{2}n+72m^{2}+6912mn^{3}+6432mn^{2}-6624mn+624m+5056n^{3}-672n^{2}-4624n+1848\right)/3.$

(ix) *Schultz*
  -
$S\left(G\right)=\left(103680m^{3}n^{2}-14400m^{3}n+480m^{3}+69120m^{2}n^{3}+253440m^{2}n^{2}-183600m^{2}n+10440m^{2}+656640mn^{4}-41760mn^{3}-21120mn^{2}-142920mn+4800m-566784n^{5}+729840n^{4}-257600n^{3}-20640n^{2}+34424n-18360\right)/15.$

(x) *Gutman*
  -
$Gut\left(G\right)=\left(138240m^{3}n^{2}-23040m^{3}n+960m^{3}+92160m^{2}n^{3}+322560m^{2}n^{2}-246480m^{2}n+16680m^{2}+875520mn^{4}-103680mn^{3}-37440mn^{2}-171120mn+6840m-755712n^{5}+992640n^{4}-382400n^{3}-12480n^{2}+52592n-27360\right)/15.$

**Figure 4:**
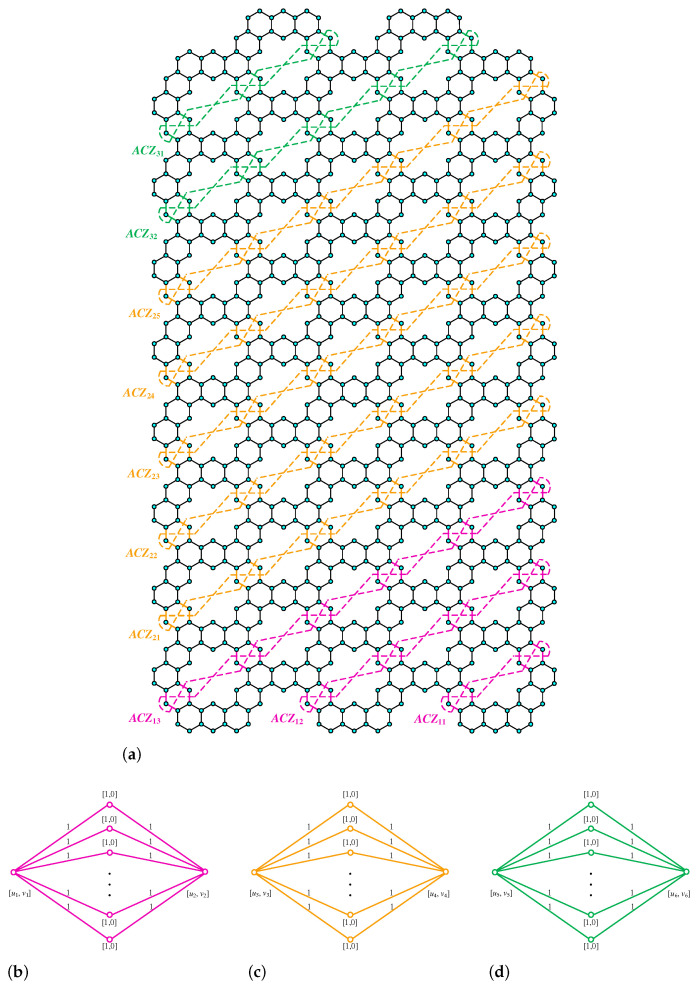
(**a**) $ACZ_{1i},ACZ_{2i},ACZ_{3i}$; (**b**) $G/ACZ_{1i},i\in \left[n\right]$; (**c**) $G/ACZ_{2i},i\in \left[m-n\right]$; (**d**) $G/ACZ_{3i},$ i∈[n−1].

**Figure 5:**
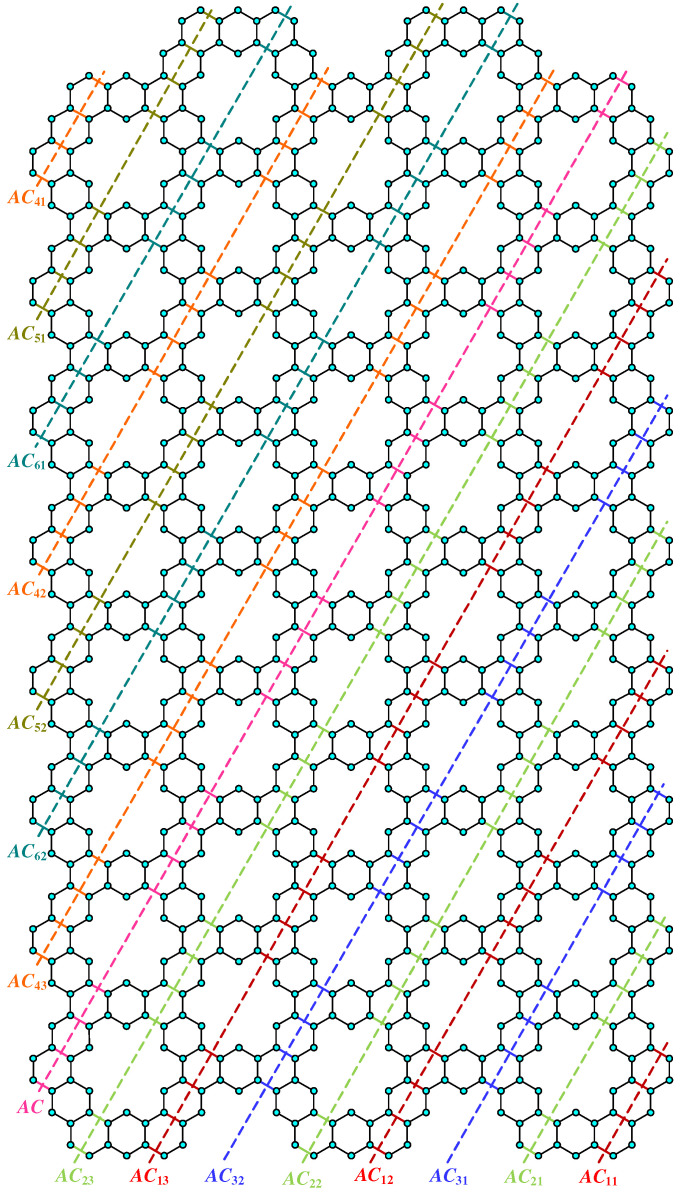
$AC_{1i}$, $AC_{2i}$, $AC_{3i}$, $AC_{4i}$, $AC_{5i}$, $AC_{6i}$, and AC.

**Figure 6:**
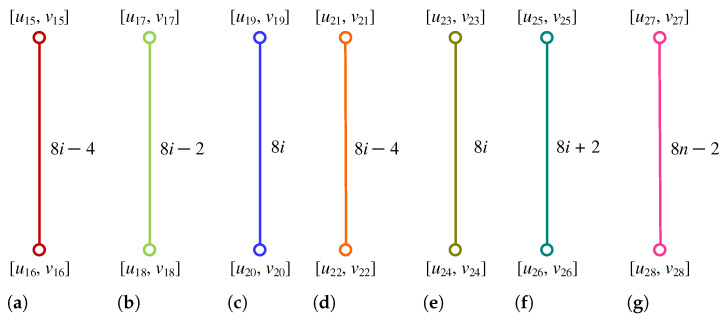
(**a**) $G/AC_{1i},i\in \left[n\right]$; (**b**) $G/AC_{2i},i\in \left[n\right]$; (**c**) $G/AC_{3i},i\in \left[n-1\right]$; (**d**) $G/AC_{4i},i\in \left[n\right]$; (**e**) $G/AC_{5i},i\in \left[n-1\right]$; (**f**) $G/AC_{6i},i\in \left[n-1\right]$; (**g**) G/AC.

**Figure 7:**
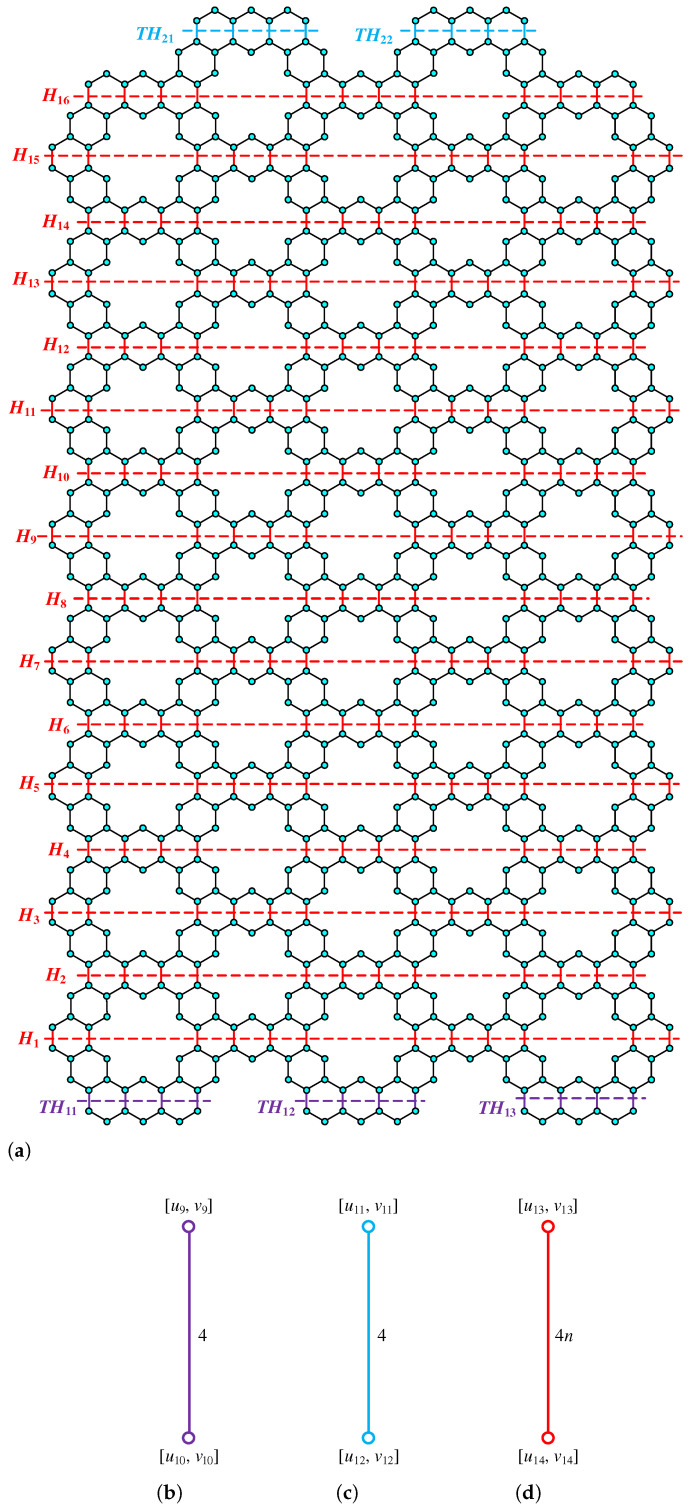
(**a**) $TH_{i}$, $H_{i}$; (**b**) $G/TH_{1i},i\in \left[n\right]$; (**c**) $G/TH_{2i},i\in \left[n-1\right]$; (**d**) $G/H_{i},i\in \left[2m\right]$.

**Figure 8:**
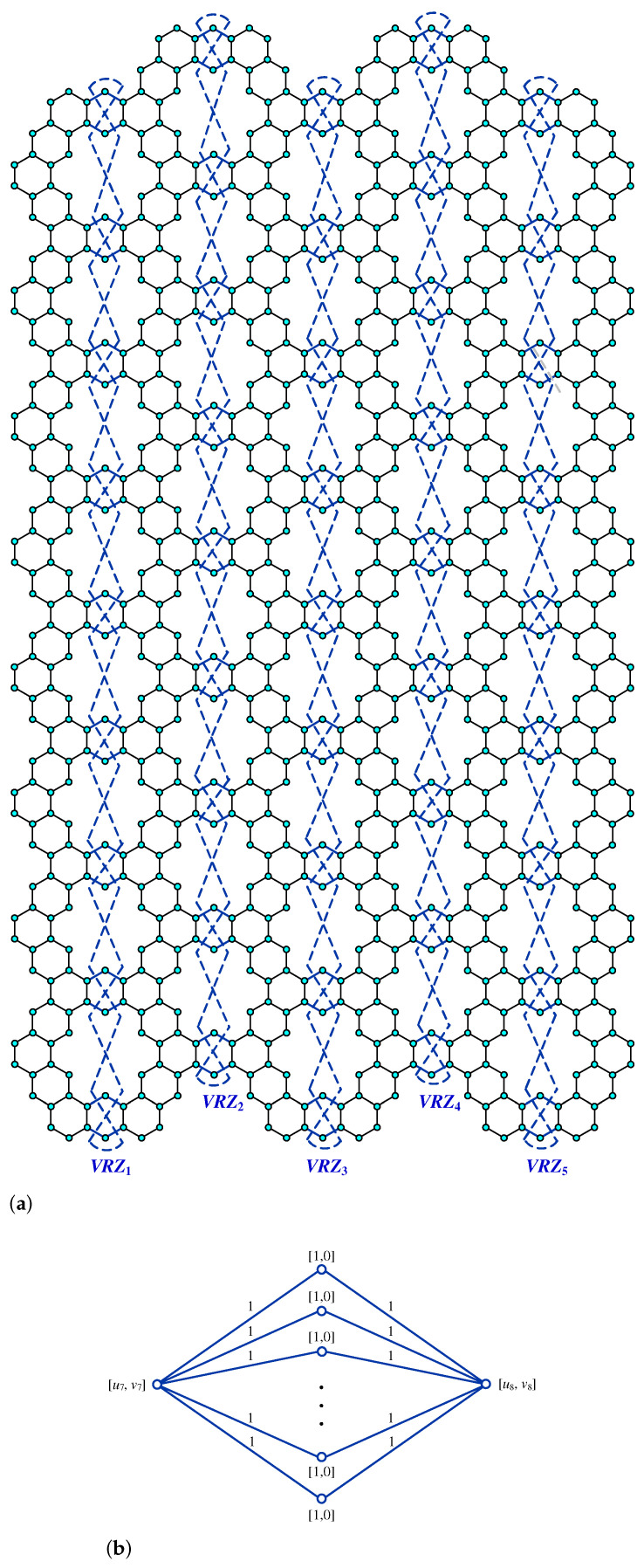
(**a**) $VRZ_{i}$; (**b**) $G/VRZ_{i},i\in \left[2n-1\right]$.

**Proof.**  We begin by describing the $\Theta^{*}$-classes for the m=3n−1 scenario. Let $\left\{ACZ_{1i}:i\in \left[n\right]\right\}$, $\left\{ACZ_{2i}:i\in \left[m-n\right]\right\}$, and $\left\{ACZ_{3i}:i\in \left[n-1\right]\right\}$ be the zigzag acute classes shown in Figure 4a, with the isomorphic SWQGs are shown in Figure 4b–d. Let $\left\{AC_{1i}:i\in \left[n\right]\right\}$, $\left\{AC_{2i}:i\in \left[n\right]\right\}$, $\left\{AC_{3i}:i\in \left[n-1\right]\right\}$, $\left\{AC_{4i}:i\in \left[n\right]\right\}$, $\left\{AC_{5i}:i\in \left[n-1\right]\right\}$, $\left\{AC_{6i}:i\in \left[n-1\right]\right\}$, and AC be acute classes shown in Figure 5 with the isomorphic SWQGs are shown in Figure 6a–g. When we rotate all of these zigzag acutes and acute classes $60^{\circ }$ anti-clockwise, we obtain zigzag obtuse $\left\{OBZ_{1i}:i\in \left[n\right]\right\}$, $\left\{OBZ_{2i}:i\in \left[m-n\right]\right\}$, $\left\{OBZ_{3i}:i\in \left[n-1\right]\right\}$ and obtuse classes $\left\{OB_{1i}:i\in \left[n\right]\right\}$, $\left\{OB_{2i}:i\in \left[n\right]\right\}$, $\left\{OB_{3i}:i\in \left[n-1\right]\right\}$, $\left\{OB_{4i}:i\in \left[n\right]\right\}$, $\left\{OB_{5i}:i\in \left[n-1\right]\right\}$, $\left\{OB_{6i}:i\in \left[n-1\right]\right\}$, and OB with no change in isomorphic SWQGs. The terminal horizontal classes and horizontal classes $\left\{TH_{1i}:i\in \left[n\right]\right\}$, $\left\{TH_{2i}:i\in \left[n-1\right]\right\}$, and $\left\{H_{i}:i\in \left[2m\right]\right\}$ are accordingly depicted in Figure 7a as well as the quotient graphs for them in Figure 7b–d. Figure 8a depicts the vertical zigzag classes $\left\{VRZ_{i}:i\in \left[2n-1\right]\right\}$ in addition to the quotient graph in Figure 8b. The above quotient graphs’ vertex strength-weighted values are shown in Table 1.

$$\begin{array}{ccc} W(G) & =2[\sum_{i\in [n]}\left[2u_{1}u_{2}+4i\left(u_{1}+u_{2}+4i-1\right)\right]+\sum_{i\in [m-n]}\left[2u_{3}u_{4}+4n\left(u_{3}+u_{4}+4n-1\right)\right] & \\ & \;\;\;+\sum_{i\in [n-1]}\left[2u_{5}u_{6}+\left(4i+2\right)\left(u_{5}+u_{6}+4i+1\right)\right]]+\sum_{i\in [2n-1]}\left[2u_{7}u_{8}+\left(2m+2\right)\left(u_{7}+u_{8}+2m+1\right)\right] & \\ & \;\;\;+\sum_{i\in [n]}u_{9}u_{10}+\sum_{i\in [n-1]}u_{11}u_{12}+\sum_{i\in [2m]}u_{13}u_{14}+2[\sum_{i\in [n]}u_{15}u_{16}+\sum_{i\in [n]}u_{17}u_{18}+\sum_{i\in [n-1]}u_{19}u_{20}+\sum_{i\in [n]}u_{21}u_{22} & \\ & \;\;\;+\sum_{i\in [n-1]}u_{23}u_{24}+\sum_{i\in [n-1]}u_{25}u_{26}]+2u_{27}u_{28}. & \end{array}$$

$$\begin{array}{ccc} W_{e}\left(G\right) & =2[\sum_{i\in [n]}\left[2v_{1}v_{2}+4i\left(v_{1}+v_{2}+4i-1\right)\right]+\sum_{i\in [m-n]}\left[2v_{3}v_{4}+4n\left(v_{3}+v_{4}+4n-1\right)\right] & \\ & \;\;\;+\sum_{i\in [n-1]}\left[2v_{5}v_{6}+\left(4i+2\right)\left(v_{5}+v_{6}+4i+1\right)\right]]+\sum_{i\in [2n-1]}\left[2v_{7}v_{8}+\left(2m+2\right)\left(v_{7}+v_{8}+2m+1\right)\right] & \\ & \;\;\;+\sum_{i\in [n]}v_{9}v_{10}+\sum_{i\in [n-1]}v_{11}v_{12}+\sum_{i\in [2m]}v_{13}v_{14}+2[\sum_{i\in [n]}v_{15}v_{16}+\sum_{i\in [n]}v_{17}v_{18}+\sum_{i\in [n-1]}v_{19}v_{20}+\sum_{i\in [n]}v_{21}v_{22} & \\ & \;\;\;+\sum_{i\in [n-1]}v_{23}v_{24}+\sum_{i\in [n-1]}v_{25}v_{26}]+2v_{27}v_{28}. & \end{array}$$

$$\begin{array}{ccc} W_{ve}\left(G\right) & =\frac{1}{2}[2[\sum_{i\in [n]}\left[4i\left(u_{1}+u_{2}+v_{1}+v_{2}+8i-2\right)+2\left(u_{1}v_{2}+u_{2}v_{1}\right)\right] & \\ & \;\;\;+\sum_{i\in [m-n]}\left[4n\left(u_{3}+u_{4}+v_{3}+v_{4}+8n-2\right)+2\left(u_{3}v_{4}+u_{4}v_{3}\right)\right] & \\ & \;\;\;+\sum_{i\in [n-1]}\left[\left(4i+2\right)\left(u_{5}+u_{6}+v_{5}+v_{6}+8i+2\right)+2\left(u_{5}v_{6}+u_{6}v_{5}\right)\right]] & \\ & \;\;\;+\sum_{i\in [2n-1]}\left[\left(2m+2\right)\left(u_{7}+u_{8}+v_{7}+v_{8}+4m+2\right)+2\left(u_{7}v_{8}+u_{8}v_{7}\right)\right]+\sum_{i\in [n]}\left[u_{9}v_{10}+u_{10}v_{9}\right] & \\ & \;\;\;+\sum_{i\in [n-1]}\left[u_{11}v_{12}+u_{12}v_{11}\right]+\sum_{i\in [2m]}\left[u_{13}v_{14}+u_{14}v_{13}\right]+2[\sum_{i\in [n]}\left[u_{15}v_{16}+u_{16}v_{15}\right]+\sum_{i\in [n]}\left[u_{17}v_{18}+u_{18}v_{17}\right] & \\ & \;\;\;+\sum_{i\in [n-1]}\left[u_{19}v_{20}+u_{20}v_{19}\right]+\sum_{i\in [n]}\left[u_{21}v_{22}+u_{22}v_{21}\right]+\sum_{i\in [n-1]}\left[u_{23}v_{24}+u_{24}v_{23}\right]+\sum_{i\in [n-1]}\left[u_{25}v_{26}+u_{26}v_{25}\right]] & \\ & \;\;\;+2(u_{27}v_{28}+u_{28}v_{27})]. & \end{array}$$

$$\begin{array}{ccc} Sz_{v}\left(G\right) & =2[\sum_{i\in [n]}4i\left(\left(u_{1}+4i-1\right)\left(u_{2}+1\right)+\left(u_{2}+4i-1\right)\left(u_{1}+1\right)\right) & \\ & \;\;\;+\sum_{i\in [m-n]}4n\left(\left(u_{3}+4n-1\right)\left(u_{4}+1\right)+\left(u_{4}+4n-1\right)\left(u_{3}+1\right)\right) & \\ & \;\;\;+\sum_{i\in [n-1]}\left(4i+2\right)\left(\left(u_{5}+4i+1\right)\left(u_{6}+1\right)+\left(u_{6}+4i+1\right)\left(u_{5}+1\right)\right)] & \\ & \;\;\;+\sum_{i\in [2n-1]}\left(2m+2\right)\left(\left(u_{7}+2m+1\right)\left(u_{8}+1\right)+\left(u_{8}+2m+1\right)\left(u_{7}+1\right)\right)+\sum_{i\in [n]}4u_{9}u_{10}+\sum_{i\in [n-1]}4u_{11}u_{12} & \\ & \;\;\;+\sum_{i\in [2m]}4nu_{13}u_{14}+2[\sum_{i\in [n]}\left(8i-4\right)u_{15}u_{16}+\sum_{i\in [n]}\left(8i-2\right)u_{17}u_{18}+\sum_{i\in [n-1]}8iu_{19}u_{20}+\sum_{i\in [n]}\left(8i-4\right)u_{21}u_{22} & \\ & \;\;\;+\sum_{i\in [n-1]}8iu_{23}u_{24}+\sum_{i\in [n-1]}\left[\left(8i+2\right)u_{25}u_{26}\right]]+2\left(8n-2\right)u_{27}u_{28}. & \end{array}$$

$$\begin{array}{ccc} Sz_{e}\left(G\right) & =2[\sum_{i\in [n]}4i\left(\left(v_{1}+4i-1\right)\left(v_{2}+1\right)+\left(v_{2}+4i-1\right)\left(v_{1}+1\right)\right) & \\ & \;\;\;+\sum_{i\in [m-n]}4n\left(\left(v_{3}+4n-1\right)\left(v_{4}+1\right)+\left(v_{4}+4n-1\right)\left(v_{3}+1\right)\right) & \\ & \;\;\;+\sum_{i\in [n-1]}\left(4i+2\right)\left(\left(v_{5}+4i+1\right)\left(v_{6}+1\right)+\left(v_{6}+4i+1\right)\left(v_{5}+1\right)\right)] & \\ & \;\;\;+\sum_{i\in [2n-1]}\left(2m+2\right)\left(\left(v_{7}+2m+1\right)\left(v_{8}+1\right)+\left(v_{8}+2m+1\right)\left(v_{7}+1\right)\right)+\sum_{i\in [n]}4v_{9}v_{10}+\sum_{i\in [n-1]}4v_{11}v_{12} & \\ & \;\;\;+\sum_{i\in [2m]}4nv_{13}v_{14}+2[\sum_{i\in [n]}\left(8i-4\right)v_{15}v_{16}+\sum_{i\in [n]}\left(8i-2\right)v_{17}v_{18}+\sum_{i\in [n-1]}8iv_{19}v_{20}+\sum_{i\in [n]}\left(8i-4\right)v_{21}v_{22} & \\ & \;\;\;+\sum_{i\in [n-1]}8iv_{23}v_{24}+\sum_{i\in [n-1]}\left(8i+2\right)v_{25}v_{26}]+2\left(8n-2\right)v_{27}v_{28}. & \end{array}$$

$$\begin{array}{ccc} Sz_{ev}\left(G\right) & =\frac{1}{2}[2[\sum_{i\in [n]}4i\left(2u_{1}v_{2}+4iv_{2}+16i+2v_{1}u_{2}+4iu_{2}+4iv_{1}+4iu_{1}-4\right) & \\ & \;\;\;+\sum_{i\in [m-n]}4n\left(2u_{3}v_{4}+4nv_{4}+16n+2v_{3}u_{4}+4nu_{4}+4nv_{3}+4nu_{3}-4\right) & \\ & \;\;\;+\sum_{i\in [n-1]}2\left(2i+1\right)\left(2u_{5}v_{6}+2u_{5}+4iv_{6}+16i+2v_{6}+2v_{5}u_{6}+2v_{5}+4iu_{6}+2u_{6}+4iv_{5}+4iu_{5}+4\right)] & \\ & \;\;\;+\sum_{i\in [2n-1]}\left(2m+2\right)\left(2u_{7}v_{8}+2u_{7}+2mv_{8}+8m+2v_{8}+2v_{7}u_{8}+2v_{7}+2mu_{8}+2u_{8}+2mv_{7}+2mu_{7}+4\right) & \\ & \;\;\;+\sum_{i\in [n]}4\left(u_{9}v_{10}+u_{10}v_{9}\right)+\sum_{i\in [n-1]}4\left(u_{11}v_{12}+u_{12}v_{11}\right)+\sum_{i\in [2m]}4n\left(u_{13}v_{14}+u_{14}v_{13}\right) & \\ & \;\;\;+2[\sum_{i\in [n]}\left(8i-4\right)\left(u_{15}v_{16}+u_{16}v_{15}\right)+\sum_{i\in [n]}\left(8i-2\right)\left(u_{17}v_{18}+u_{18}v_{17}\right)+\sum_{i\in [n-1]}8i\left(u_{19}v_{20}+u_{20}v_{19}\right) & \\ & \;\;\;+\sum_{i\in [n]}\left(8i-4\right)\left(u_{21}v_{22}+u_{22}v_{21}\right)+\sum_{i\in [n-1]}8i\left(u_{23}v_{24}+u_{24}v_{23}\right)+\sum_{i\in [n-1]}\left[\left(8i+2\right)\left(u_{25}v_{26}+u_{26}v_{25}\right)\right]] & \\ & \;\;\;+2\left(8n-2\right)\left(u_{27}v_{28}+u_{28}v_{27}\right)]. & \end{array}$$

$$\begin{array}{ccc} Sz_{t}\left(G\right) & =Sz_{v}\left(G\right)+Sz_{e}\left(G\right)+2Sz_{ev}\left(G\right). & \end{array}$$

$$\begin{array}{ccc} PI(G) & =2\left[\sum_{i\in [n]}8i\left(v_{1}+v_{2}+4i\right)+\sum_{i\in [m-n]}8n\left(v_{3}+v_{4}+4n\right)+\sum_{i\in [n-1]}\left(8i+4\right)\left(v_{5}+v_{6}+4i+2\right)\right] & \\ & \;\;\;+\sum_{i\in [2n-1]}\left(4m+4\right)\left(v_{7}+v_{8}+2m+2\right)+\sum_{i\in [n]}4\left(v_{9}+v_{10}\right)+\sum_{i\in [n-1]}4\left(v_{11}+v_{12}\right)+\sum_{i\in [2m]}4n\left(v_{13}+v_{14}\right) & \\ & \;\;\;+2[\sum_{i\in [n]}\left(8i-4\right)\left(v_{15}+v_{16}\right)+\sum_{i\in [n]}\left(8i-2\right)\left(v_{17}+v_{18}\right)+\sum_{i\in [n-1]}8i\left(v_{19}+v_{20}\right)+\sum_{i\in [n]}\left(8i-4\right)\left(v_{21}+v_{22}\right) & \\ & \;\;\;+\sum_{i\in [n-1]}8i\left(v_{23}+v_{24}\right)+\sum_{i\in [n-1]}\left(8i+2\right)\left(v_{25}+v_{26}\right)]+2\left(8n-2\right)\left(v_{27}+v_{28}\right). & \end{array}$$

$$\begin{array}{ccc} S(G) & =2[\sum_{i\in [n]}\left[96i^{2}+16iu_{1}+16iu_{2}+8iv_{1}+8iv_{2}+4u_{1}v_{2}-16i+4u_{2}v_{1}\right] & \\ & \;\;\;+\sum_{i\in [m-n]}\left[96n^{2}+16nu_{3}+16nu_{4}+8nv_{3}+8nv_{4}+4u_{3}v_{4}-16n+4u_{4}v_{3}\right] & \\ & \;\;\;+\sum_{i\in [n-1]}\left[96i^{2}+8u_{5}+80i+8u_{6}+4v_{5}+4v_{6}+16iu_{5}+16iu_{6}+8iv_{5}+8iv_{6}+4u_{5}v_{6}+4u_{6}v_{5}+16\right]] & \\ & \;\;\;+\sum_{i\in [2n-1]}\left[24m^{2}+40m+8u_{7}+8u_{8}+4v_{7}+4v_{8}+8mu_{7}+8mu_{8}+4mv_{7}+4mv_{8}+4u_{7}v_{8}+4u_{8}v_{7}+16\right] & \\ & \;\;\;+\sum_{i\in [n]}\left[u_{9}\left(2v_{10}+4\right)+u_{10}\left(2v_{9}+4\right)\right]+\sum_{i\in [n-1]}\left[u_{11}\left(2v_{12}+4\right)+u_{12}\left(2v_{11}+4\right)\right] & \\ & \;\;\;+\sum_{i\in [2m]}\left[u_{13}\left(2v_{14}+4n\right)+u_{14}\left(2v_{13}+4n\right)\right]+2[\sum_{i\in [n]}\left[u_{15}\left(2v_{16}+8i-4\right)+u_{16}\left(2v_{15}+8i-4\right)\right] & \\ & \;\;\;+\sum_{i\in [n]}\left[u_{17}\left(2v_{18}+8i-2\right)+u_{18}\left(2v_{17}+8i-2\right)\right]+\sum_{i\in [n-1]}\left[u_{19}\left(2v_{20}+8i\right)+u_{20}\left(2v_{19}+8i\right)\right] & \\ & \;\;\;+\sum_{i\in [n]}\left[u_{21}\left(2v_{22}+8i-4\right)+u_{22}\left(2v_{21}+8i-4\right)\right]+\sum_{i\in [n-1]}\left[u_{23}\left(2v_{24}+8i\right)+u_{24}\left(2v_{23}+8i\right)\right] & \\ & \;\;\;+\sum_{i\in [n-1]}\left[u_{25}\left(2v_{26}+8i+2\right)+u_{26}\left(2v_{25}+8i+2\right)\right]]+2\left[u_{27}\left(2v_{28}+8n-2\right)+u_{28}\left(2v_{27}+8n-2\right)\right]. & \end{array}$$

$$\begin{array}{ccc} Gut(G) & =2[\sum_{i\in [n]}\left[8i\left(2\left(v_{1}+v_{2}\right)+8i\right)+2\left[\left(2v_{1}+4i\right)\left(2v_{2}+4i\right)\right]+16i\left(4i-1\right)\right] & \\ & \;\;\;+\sum_{i\in [m-n]}\left[8n\left(2\left(v_{3}+v_{4}\right)+8n\right)+2\left[\left(2v_{3}+4n\right)\left(2v_{4}+4n\right)\right]+16n\left(4n-1\right)\right] & \\ & \;\;\;+\sum_{i\in [n-1]}\left[\left(8i+4\right)\left(2\left(v_{5}+v_{6}\right)+8i+4\right)+2\left[\left(2v_{5}+4i+2\right)\left(2v_{6}+4i+2\right)\right]+4\left(4i+2\right)\left(4i+1\right)\right]] & \\ & \;\;\;+\sum_{i\in [2n-1]}\left[\left(4m+4\right)\left(2\left(v_{7}+v_{8}\right)+4m+4\right)+2\left[\left(2v_{7}+2m+2\right)\left(2v_{8}+2m+2\right)\right]+4\left(2m+2\right)\left(2m+1\right)\right] & \\ & \;\;\;+\sum_{i\in [n]}\left[\left(2v_{9}+4\right)\left(2v_{10}+4\right)\right]+\sum_{i\in [n-1]}\left[\left(2v_{11}+4\right)\left(2v_{12}+4\right)\right]+\sum_{i\in [2m]}\left[\left(2v_{13}+4n\right)\left(2v_{14}+4n\right)\right] & \\ & \;\;\;+2[\sum_{i\in [n]}\left[\left(2v_{15}+8i-4\right)\left(2v_{16}+8i-4\right)\right]+\sum_{i\in [n]}\left[\left(2v_{17}+8i-2\right)\left(2v_{18}+8i-2\right)\right] & \\ & \;\;\;+\sum_{i\in [n-1]}\left[\left(2v_{19}+8i\right)\left(2v_{20}+8i\right)\right]+\sum_{i\in [n]}\left[\left(2v_{21}+8i-4\right)\left(2v_{22}+8i-4\right)\right]+\sum_{i\in [n-1]}\left[\left(2v_{23}+8i\right)\left(2v_{24}+8i\right)\right] & \\ & \;\;\;+\sum_{i\in [n-1]}\left[\left(2v_{25}+8i+2\right)\left(2v_{26}+8i+2\right)\right]]+2\left(2v_{27}+8n-2\right)\left(2v_{28}+8n-2\right). & \end{array}$$

By substituting the Table 1 values, the proof is complete by just simplifying it. □

**Theorem** **2.** *Let G be a RK(m,n) Type-I rectangular kekulene system with m>3n−1.*

(i) *Wiener*
  - *$W\left(G\right)=\left(25920m^{3}n^{2}-2880m^{3}n+80m^{3}+12960m^{2}n^{3}+66960m^{2}n^{2}-44760m^{2}n+2100m^{2}+35640mn^{4}+15120mn^{3}+39090mn^{2}-44730mn+1285m-18792n^{5}+36900n^{4}-29000n^{3}+8280n^{2}+1652n-3015\right)/15$.*
(ii) *Edge-Wiener*
  -
$W_{e}\left(G\right)=\left(46080m^{3}n^{2}-7680m^{3}n+320m^{3}+23040m^{2}n^{3}+92160m^{2}n^{2}-78360m^{2}n+5460m^{2}+63360mn^{4}+17280mn^{3}+28320mn^{2}-50100mn+1570m-33408n^{5}+66720n^{4}-57640n^{3}+3600n^{2}+22048n-11310\right)/15.$

(iii) *Vertex-Edge-Wiener*
  -
$W_{ve}\left(G\right)=\left(43200m^{3}n^{2}-6000m^{3}n+200m^{3}+17280m^{2}n^{3}+99360m^{2}n^{2}-73920m^{2}n+4380m^{2}-69120mn^{4}+50040mn^{3}+83640mn^{2}-67950mn+2470m+91584n^{5}-83220n^{4}-11480n^{3}+17400n^{2}+6146n-6210\right)/15.$

(iv) *Vertex-Szeged*
  -
$Sz_{v}\left(G\right)=\left(155520m^{3}n^{3}-18720m^{3}n^{2}-3920m^{3}n+417600m^{2}n^{3}-260640m^{2}n^{2}+18480m^{2}n-1080m^{2}-17280mn^{4}+389720mn^{3}-324240mn^{2}+68460mn-7380m+6624n^{5}-49720n^{4}+107280n^{3}-96320n^{2}+28476n-4620\right)/15.$

(v) *Edge-Szeged*
  -
$Sz_{e}\left(G\right)=\left(276480m^{3}n^{3}-55680m^{3}n^{2}-320m^{3}n-480m^{3}+618240m^{2}n^{3}-493440m^{2}n^{2}+61680m^{2}n-5160m^{2}+30720mn^{4}+490720mn^{3}-523680mn^{2}+164200mn-20100m+10752n^{5}-25920n^{4}+70560n^{3}-125520n^{2}+76608n-21540\right)/15.$

(vi) *Edge-Vertex-Szeged*
  -
$Sz_{ev}\left(G\right)=\left(276480m^{3}n^{3}-42960m^{3}n^{2}-2600m^{3}n-120m^{3}+671520m^{2}n^{3}-475440m^{2}n^{2}+42480m^{2}n-2520m^{2}-933120mn^{5}+267840mn^{4}+822960mn^{3}-567000mn^{2}+114200mn-12180m+933120n^{6}-1054272n^{5}+195320n^{4}+183920n^{3}-138800n^{2}+47932n-11040\right)/15.$

(vii) *Total-Szeged*
  -
$Sz_{t}\left(G\right)=\left(984960m^{3}n^{3}-160320m^{3}n^{2}-9440m^{3}n-720m^{3}+2378880m^{2}n^{3}-1704960m^{2}n^{2}+165120m^{2}n-11280m^{2}-1866240mn^{5}+549120mn^{4}+2526360mn^{3}-1981920mn^{2}+461060mn-51840m+1866240n^{6}-2091168n^{5}+315000n^{4}+545680n^{3}-499440n^{2}+200948n-48240\right)/15.$

(viii) *Padmakar–Ivan*
  -
$PI\left(G\right)=\left(6912m^{2}n^{2}-1200m^{2}n+72m^{2}+10848mn^{2}-7968mn+624m+448n^{3}+4320n^{2}-5776n+1848\right)/3.$

(ix) *Schultz*
  -
$S\left(G\right)=\left(138240m^{3}n^{2}-19200m^{3}n+640m^{3}+69120m^{2}n^{3}+342720m^{2}n^{2}-241680m^{2}n+14040m^{2}+190080mn^{4}+66240mn^{3}+187920mn^{2}-224280mn+8240m-100224n^{5}+198480n^{4}-163280n^{3}+44400n^{2}+16184n-18360\right)/15.$

(x) *Gutman*
  -
$Gut\left(G\right)=\left(184320m^{3}n^{2}-30720m^{3}n+1280m^{3}+92160m^{2}n^{3}+437760m^{2}n^{2}-325200m^{2}n+22440m^{2}+253440mn^{4}+69120mn^{3}+225120mn^{2}-279600mn+12280m-133632n^{5}+266880n^{4}-228320n^{3}+60000n^{2}+30512n-27360\right)/15.$

**Proof.**  With regard to the m>3n−1 case, all of these $\Theta^{*}$-classes, as in the m=3n−1 case, exist. We also have a few other classes as follows: $\left\{AC_{7i}:i\in \left[m-3n+1\right]\right\}$ and $\left\{OB_{4i}:i\in \left[m-3n+1\right]\right\}$. The SWQG’s vertex values of $AC_{7i}$ are $u_{29}=54n^{2}+36ni-14n-2i-16$, $u_{30}=\left|V\right|-u_{29}$, $v_{29}=72n^{2}+48ni-26n-4i-20$, and $v_{30}=\left|E\right|-v_{29}-8n$ and its edge strength is 8n. Additionally, it is simple to see the isomorphism between SWQGs of $AC_{7i}$ and $OB_{7i}$.

By applying Theorem 1’s justifications and substituting Table 1’s values, the proof is complete by simplifying it. □

**Theorem** **3.** *Let G be a RK(m,n) Type-II rectangular kekulene system with m=3n−1.*

(i) *Wiener*
  - *$W\left(G\right)=\left(19440m^{3}n^{2}-2160m^{3}n+60m^{3}+8480m^{2}n^{3}+95220m^{2}n^{2}-82640m^{2}n+9060m^{2}+123120mn^{4}+31100mn^{3}+47490mn^{2}-153170mn+42465m-106272n^{5}+151920n^{4}-30500n^{3}-42645n^{2}-10648n+19770\right)/15$.*
(ii) *Edge-Wiener*
  -
$W_{e}\left(G\right)=\left(34560m^{3}n^{2}-5760m^{3}n+240m^{3}+23040m^{2}n^{3}+120960m^{2}n^{2}-115320m^{2}n+8340m^{2}+218880mn^{4}+31680mn^{3}-24360mn^{2}-154860mn+52410m-188928n^{5}+279840n^{4}-92320n^{3}-106500n^{2}+69538n+300\right)/15.$

(iii) *Vertex-Edge-Wiener*
  -
$W_{ve}\left(G\right)=\left(25920m^{3}n^{2}-3600m^{3}n+120m^{3}+15600m^{2}n^{3}+102960m^{2}n^{2}-90060m^{2}n+5280m^{2}+164160mn^{4}+35320mn^{3}+9660mn^{2}-143260mn+40440m-141696n^{5}+206220n^{4}-54340n^{3}-75180n^{2}+28216n+9480\right)/15.$

(iv) *Vertex-Szeged*
  -
$Sz_{v}\left(G\right)=\left(94720m^{3}n^{3}+19800m^{3}n^{2}-39640m^{3}n+11760m^{3}+397080m^{2}n^{3}-204600m^{2}n^{2}-120240m^{2}n+45000m^{2}+699840mn^{5}+86400mn^{4}+121360mn^{3}-581040mn^{2}+107060mn+32460m-699840n^{6}+784224n^{5}+157280n^{4}-370940n^{3}-178880n^{2}+225356n-40680\right)/15.$

(v) *Edge-Szeged*
  -
$Sz_{e}\left(G\right)=\left(184320m^{3}n^{3}-40320m^{3}n^{2}-960m^{3}n-480m^{3}+710400m^{2}n^{3}-653760m^{2}n^{2}+92640m^{2}n-10200m^{2}+1244160mn^{5}-38400mn^{4}+146080mn^{3}-1260480mn^{2}+626600mn-69540m-1244160n^{6}+1462272n^{5}+72960n^{4}-643760n^{3}-297600n^{2}+577748n-167160\right)/15.$

(vi) *Edge-Vertex-Szeged*
  -
$Sz_{ev}\left(G\right)=\left(269760m^{3}n^{3}-27360m^{3}n^{2}-19920m^{3}n-240m^{3}+1083520m^{2}n^{3}-859920m^{2}n^{2}+45200m^{2}n-9840m^{2}+1866240mn^{5}+86400mn^{4}+292320mn^{3}-1843680mn^{2}+785760mn-85800m-1866240n^{6}+2142336n^{5}+256720n^{4}-971360n^{3}-507400n^{2}+804224n-210240\right)/15.$

(vii) *Total-Szeged*
  -
$Sz_{t}\left(G\right)=\left(818560m^{3}n^{3}-75240m^{3}n^{2}-80440m^{3}n+10800m^{3}+3274520m^{2}n^{3}-2578200m^{2}n^{2}+62800m^{2}n+15120m^{2}+5676480mn^{5}+220800mn^{4}+852080mn^{3}-5528880mn^{2}+2305180mn-208680m-5676480n^{6}+6531168n^{5}+743680n^{4}-2957420n^{3}-1491280n^{2}+2411552n-628320\right)/15.$

(viii) *Padmakar–Ivan*
  -
$PI\left(G\right)=\left(4608m^{2}n^{2}-1008m^{2}n+72m^{2}+6912mn^{3}+12192mn^{2}-13008mn+1248m+8512n^{3}+1872n^{2}-15976n+7200\right)/3.$

(ix) *Schultz*
  -
$S\left(G\right)=\left(103680m^{3}n^{2}-14400m^{3}n+480m^{3}+62400m^{2}n^{3}+429120m^{2}n^{2}-363360m^{2}n+21240m^{2}+656640mn^{4}+167200mn^{3}+87120mn^{2}-620680mn+165360m-566784n^{5}+824880n^{4}-181360n^{3}-290640n^{2}+50944n+63840\right)/15.$

(x) *Gutman*
  -
$Gut\left(G\right)=\left(138240m^{3}n^{2}-23040m^{3}n+960m^{3}+92160m^{2}n^{3}+529920m^{2}n^{2}-471120m^{2}n+33960m^{2}+875520mn^{4}+195840mn^{3}+25920mn^{2}-748800mn+221640m-755712n^{5}+1119360n^{4}-280640n^{3}-405120n^{2}+117632n+71760\right)/15.$

**Figure 9:**
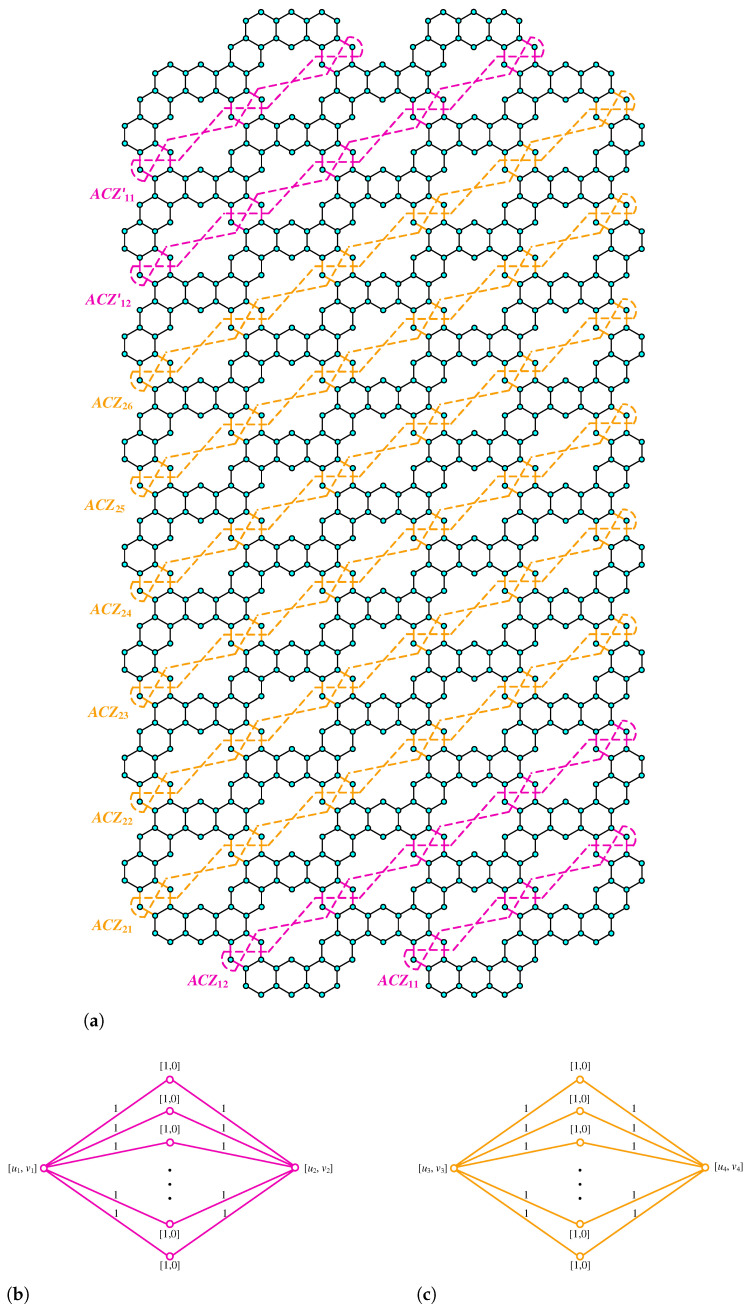
(**a**) $ACZ_{1i},ACZ_{2i}$; (**b**) $G/ACZ_{1i},i\in \left[n-1\right]$; (**c**) $G/ACZ_{2i},i\in \left[m-n+1\right]$.

**Figure 10:**
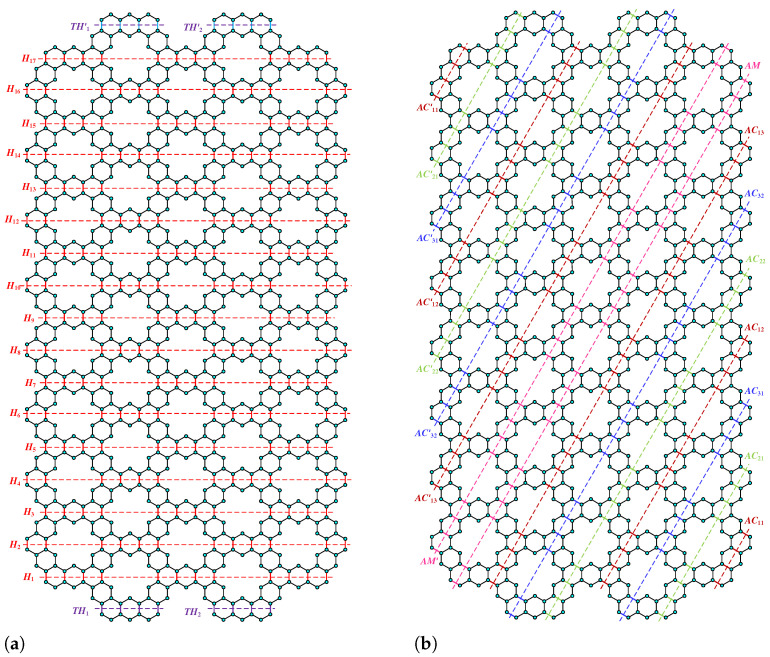
(**a**) $TH_{i}$, $H_{i}$, (**b**) $AC_{1i}$, $AC_{2i}$, $AC_{3i}$, AM.

**Figure 11:**
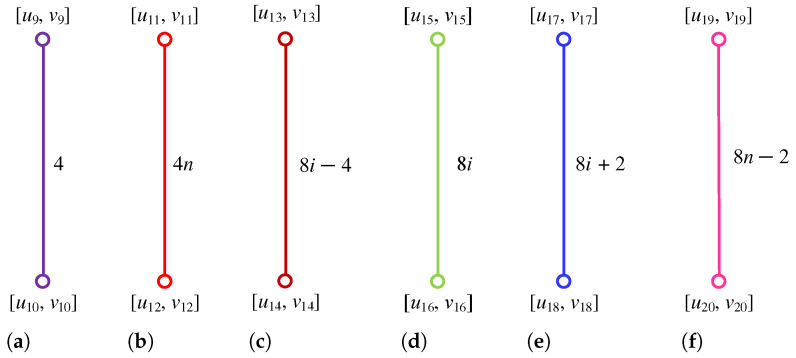
(**a**) $G/TH_{i},i\in \left[n-1\right]$; (**b**) $G/H_{i},i\in \left[2m+1\right]$; (**c**) $G/AC_{1i},i\in \left[n\right]$; (**d**) $G/AC_{2i},i\in \left[n-1\right]$; (**e**) $G/AC_{3i},i\in \left[n-1\right]$; (**f**) G/AM.

**Figure 12:**
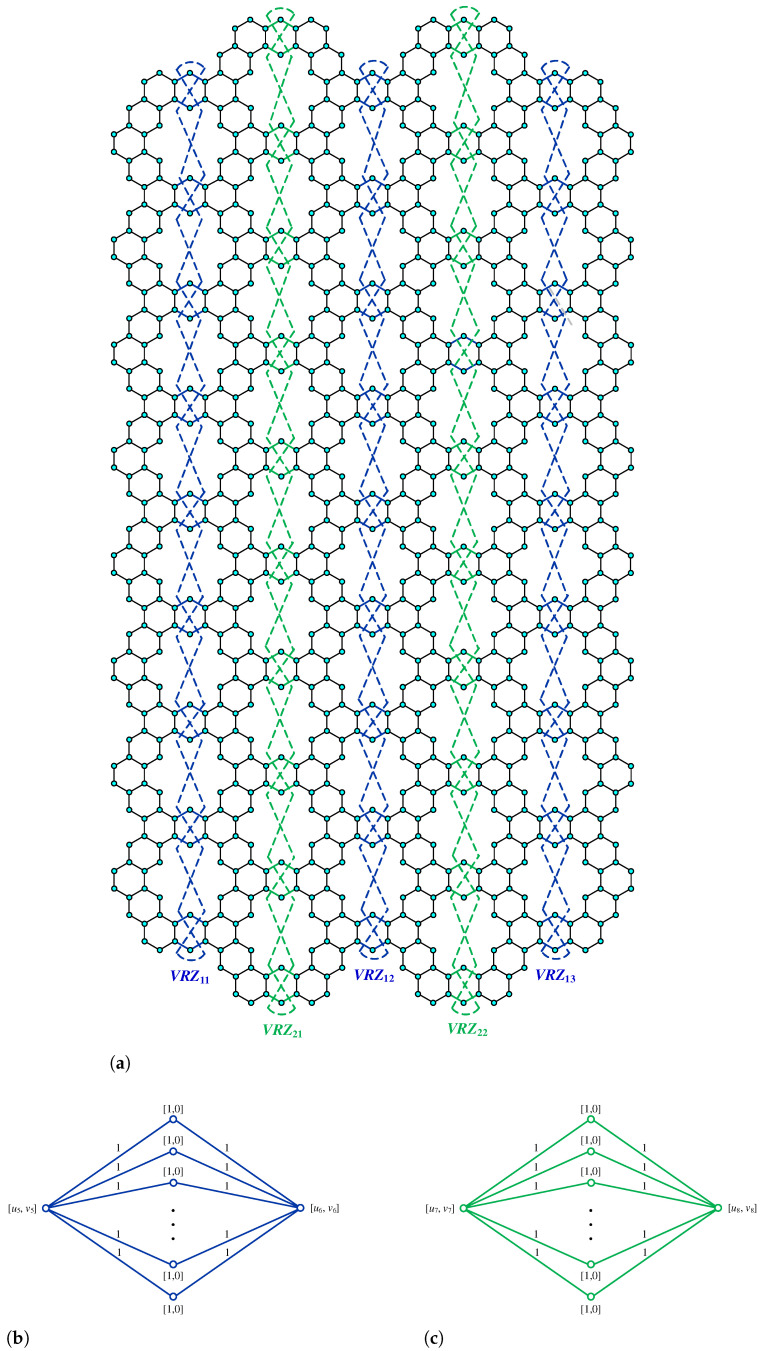
(**a**) $VRZ_{1i},VRZ_{2i}$; (**b**) $G/VRZ_{1i},i\in \left[n\right]$; (**c**) $G/VRZ_{2i},i\in \left[n-1\right]$.

**Proof.**  We begin by describing the $\Theta^{*}$-classes for the m=3n−1 scenario. Let $\left\{ACZ_{1i}:i\in \left[n-1\right]\right\}$, $\left\{ACZ_{1i}^{\prime }:i\in \left[n-1\right]\right\}$, and $\left\{ACZ_{2i}:i\in \left[m-n+1\right]\right\}$ be zigzag acute classes shown in Figure 9a with isomorphic SWQGs shown in Figure 9b,c. It is evident from the figure that $ACZ_{1i}$ and $ACZ_{1i}^{\prime }$ are similar. The terminal horizontal classes and horizontal classes $\left\{TH_{i}:i\in \left[n-1\right]\right\}$, $\left\{TH_{i}^{\prime }:i\in \left[n-1\right]\right\}$, and $\left\{H_{i}:i\in \left[2m+1\right]\right\}$ are accordingly depicted in Figure 10a and let $\left\{AC_{1i}:i\in \left[n\right]\right\}$, $\left\{AC_{2i}:i\in \left[n-1\right]\right\}$, $\left\{AC_{3i}:i\in \left[n-1\right]\right\}$, $\left\{AC_{1i}^{\prime }:i\in \left[n\right]\right\}$, $\left\{AC_{2i}^{\prime }:i\in \left[n-1\right]\right\}$, $\left\{AC_{3i}^{\prime }:i\in \left[n-1\right]\right\}$, AM, and $AM^{\prime }$ be the acute classes as shown in Figure 10b and the quotient graphs for them in Figure 11a,b and Figure 11c–f respectively. As these zigzag acute and acute classes rotate $60^{\circ }$ anti-clockwise, we obtain zigzag obtuse $\left\{OBZ_{1i}:i\in \left[n-1\right]\right\}$, $\left\{OBZ_{1i}^{\prime }:i\in \left[n-1\right]\right\}$, $\left\{OBZ_{2i}:i\in \left[m-n+1\right]\right\}$ and obtuse classes $\left\{OB_{1i}:i\in \left[n\right]\right\}$, $\left\{OB_{2i}:i\in \left[n-1\right]\right\}$, $\left\{OB_{3i}:i\in \left[n-1\right]\right\}$, $\left\{OB_{1i}^{\prime }:i\in \left[n\right]\right\}$, $\left\{OB_{2i}^{\prime }:i\in \left[n-1\right]\right\}$, $\left\{OB_{3i}^{\prime }:i\in \left[n-1\right]\right\}$, OM, and $OM^{\prime }$, with no change in isomorphic SWQGs. Figure 12a depicts the zigzag vertical classes of two varieties–that is, $\left\{VRZ_{1i}:i\in \left[n\right]\right\}$ and $\left\{VRZ_{2i}:i\in \left[n-1\right]\right\}$, and their quotient graphs, which are illustrated in Figure 12b,c. The above quotient graphs’ vertex strength-weighted values are shown in Table 2.

$$\begin{array}{ccc} W(G) & =2\left[2\sum_{i\in [n-1]}\left[2u_{1}u_{2}+\left(4i+2\right)\left(u_{1}+u_{2}+4i+1\right)\right]+\sum_{i\in [m-n-1]}\left[2u_{3}u_{4}+4n\left(u_{3}+u_{4}+4n-1\right)\right]\right] & \\ & \;\;\;+\sum_{i\in [n]}\left[2u_{5}u_{6}+\left(2m+2\right)\left(u_{5}+u_{6}+2m+1\right)\right]+\sum_{i\in [n-1]}\left[2u_{7}u_{8}+\left(2m+4\right)\left(u_{7}+u_{8}+2m+3\right)\right] & \\ & \;\;\;+2\sum_{i\in [n-1]}u_{9}u_{10}+\sum_{i\in [2m+1]}u_{11}u_{12}+4\left[\sum_{i\in [n]}u_{13}u_{14}+\sum_{i\in [n-1]}u_{15}u_{16}+\sum_{i\in [n-1]}u_{17}u_{18}\right]+4u_{19}u_{20}. & \end{array}$$

$$\begin{array}{ccc} W_{e}\left(G\right) & =2\left[2\sum_{i\in [n-1]}\left[2v_{1}v_{2}+\left(4i+2\right)\left(v_{1}+v_{2}+4i+1\right)\right]+\sum_{i\in [m-n-1]}\left[2v_{3}v_{4}+4n\left(v_{3}+v_{4}+4n-1\right)\right]\right] & \\ & \;\;\;+\sum_{i\in [n]}\left[2v_{5}v_{6}+\left(2m+2\right)\left(v_{5}+v_{6}+2m+1\right)\right]+\sum_{i\in [n-1]}\left[2v_{7}v_{8}+\left(2m+4\right)\left(v_{7}+v_{8}+2m+3\right)\right] & \\ & \;\;\;+2\sum_{i\in [n-1]}v_{9}v_{10}+\sum_{i\in [2m+1]}v_{11}v_{12}+4\left[\sum_{i\in [n]}v_{13}v_{14}+\sum_{i\in [n-1]}v_{15}v_{16}+\sum_{i\in [n-1]}v_{17}v_{18}\right]+4v_{19}v_{20}. & \end{array}$$

$$\begin{array}{ccc} W_{ve}\left(G\right) & =\frac{1}{2}[2[2\sum_{i\in [n-1]}\left[\left(4i+2\right)\left(u_{1}+u_{2}+v_{1}+v_{2}+8i+2\right)+2\left(u_{1}v_{2}+u_{2}v_{1}\right)\right] & \\ & \;\;\;+\sum_{i\in [m-n-1]}\left[4n\left(u_{3}+u_{4}+v_{3}+v_{4}+8n-2\right)+2\left(u_{3}v_{4}+u_{4}v_{3}\right)\right]] & \\ & \;\;\;+\sum_{i\in [n]}\left[\left(2m+2\right)\left(u_{5}+u_{6}+v_{5}+v_{6}+4m+2\right)+2\left(u_{5}v_{6}+u_{6}v_{5}\right)\right] & \\ & \;\;\;+\sum_{i\in [n-1]}\left[\left(2m+4\right)\left(u_{7}+u_{8}+v_{7}+v_{8}+4m+6\right)+2\left(u_{7}v_{8}+u_{8}v_{7}\right)\right]+2\sum_{i\in [n-1]}\left[u_{9}v_{10}+u_{10}v_{9}\right] & \\ & \;\;\;+\sum_{i\in [2m+1]}\left[u_{11}v_{12}+u_{12}v_{11}\right]+4\left[\sum_{i\in [n]}\left[u_{13}v_{14}+u_{14}v_{13}\right]+\sum_{i\in [n-1]}\left[u_{15}v_{16}+u_{16}v_{15}\right]+\sum_{i\in [n-1]}\left[u_{17}v_{18}+u_{18}v_{17}\right]\right] & \\ & \;\;\;+4[u_{19}v_{20}+u_{20}v_{19}]]. & \end{array}$$

$$\begin{array}{ccc} Sz_{v}\left(G\right) & =2[2\sum_{i\in [n-1]}\left(4i+2\right)\left(\left(u_{1}+4i+1\right)\left(u_{2}+1\right)+\left(u_{2}+4i+1\right)\left(u_{1}+1\right)\right) & \\ & \;\;\;+\sum_{i\in [m-n-1]}4n\left(\left(u_{3}+4n-1\right)\left(u_{4}+1\right)+\left(u_{4}+4n-1\right)\left(u_{3}+1\right)\right)] & \\ & \;\;\;+\sum_{i\in [n]}\left(2m+2\right)\left(\left(u_{5}+2m+1\right)\left(u_{6}+1\right)+\left(u_{6}+2m+1\right)\left(u_{5}+1\right)\right) & \\ & \;\;\;+\sum_{i\in [n-1]}\left(2m+4\right)\left(\left(u_{7}+2m+3\right)\left(u_{8}+1\right)+\left(u_{8}+2m+3\right)\left(u_{7}+1\right)\right)+2\sum_{i\in [n-1]}4u_{9}u_{10}+\sum_{2m+1}4nu_{11}u_{12} & \\ & \;\;\;+4\left[\sum_{i\in [n]}\left(8i-4\right)u_{13}u_{14}+\sum_{i\in [n-1]}8iu_{15}u_{16}+\sum_{i\in [n-1]}\left(8i+2\right)u_{17}u_{18}\right]+4\left(8n-2\right)u_{19}u_{20}. & \end{array}$$

$$\begin{array}{ccc} Sz_{e}\left(G\right) & =2[2\sum_{i\in [n-1]}\left(4i+2\right)\left(\left(v_{1}+4i+1\right)\left(v_{2}+1\right)+\left(v_{2}+4i+1\right)\left(v_{1}+1\right)\right) & \\ & \;\;\;+\sum_{i\in [m-n-1]}4n\left(\left(v_{3}+4n-1\right)\left(v_{4}+1\right)+\left(v_{4}+4n-1\right)\left(v_{3}+1\right)\right)] & \\ & \;\;\;+\sum_{i\in [n]}\left(2m+2\right)\left(\left(v_{5}+2m+1\right)\left(v_{6}+1\right)+\left(v_{6}+2m+1\right)\left(v_{5}+1\right)\right) & \\ & \;\;\;+\sum_{i\in [n-1]}\left(2m+4\right)\left(\left(v_{7}+2m+3\right)\left(v_{8}+1\right)+\left(v_{8}+2m+3\right)\left(v_{7}+1\right)\right)+2\sum_{i\in [n-1]}4v_{9}v_{10}+\sum_{i\in [2m+1]}4nv_{11}v_{12} & \\ & \;\;\;+4\left[\sum_{i\in [n]}\left(8i-4\right)v_{13}v_{14}+\sum_{i\in [n-1]}8iv_{15}v_{16}+\sum_{i\in [n-1]}\left(8i+2\right)v_{17}v_{18}\right]+4\left(8n-2\right)v_{19}v_{20}. & \end{array}$$

$$\begin{array}{ccc} Sz_{ev}\left(G\right) & =\frac{1}{2}[2[2\sum_{i\in [n-1]}\left(4i+2\right)\left(2u_{1}v_{2}+2u_{1}+4iv_{2}+16i+2v_{2}+2v_{1}u_{2}+2v_{1}+4iu_{2}+2u_{2}+4iv_{1}+4iu_{1}+4\right) & \\ & \;\;\;+\sum_{i\in [m-n-1]}4n\left(2u_{3}v_{4}+4nv_{4}+16n+2v_{3}u_{4}+4nu_{4}+4nv_{3}+4nu_{3}-4\right)] & \\ & \;\;\;+\sum_{i\in [n]}\left(2m+2\right)\left(2u_{5}v_{6}+2u_{5}+2mv_{6}+8m+2v_{6}+2v_{5}u_{6}+2v_{5}+2mu_{6}+2u_{6}+2mv_{5}+2mu_{5}+4\right) & \\ & \;\;\;+\sum_{i\in [n-1]}\left(2m+4\right)\left(2u_{7}v_{8}+4u_{7}+2mv_{8}+8m+4v_{8}+2v_{7}u_{8}+4v_{7}+2mu_{8}+4u_{8}+2mv_{7}+2mu_{7}+12\right) & \\ & \;\;\;+2\sum_{i\in [n-1]}4\left(u_{9}v_{10}+u_{10}v_{9}\right)+\sum_{i\in [2m+1]}4n\left(u_{11}v_{12}+u_{12}v_{11}\right)+4[\sum_{i\in [n]}\left(8i-4\right)\left(u_{13}v_{14}+u_{14}v_{13}\right) & \\ & \;\;\;+\sum_{i\in [n-1]}8i\left(u_{15}v_{16}+u_{16}v_{15}\right)+\sum_{i\in [n-1]}\left[\left(8i+2\right)\left(u_{17}v_{18}+u_{18}v_{17}\right)\right]]+4\left(8n-2\right)\left(u_{19}v_{20}+u_{20}v_{19}\right)]. & \end{array}$$

$$\begin{array}{ccc} Sz_{t}\left(G\right) & =Sz_{v}\left(G\right)+Sz_{e}\left(G\right)+2Sz_{ev}\left(G\right). & \end{array}$$

$$\begin{array}{ccc} PI(G) & =2\left[2\sum_{i\in [n-1]}\left(8i+4\right)\left(v_{1}+v_{2}+4i+2\right)+\sum_{i\in [m-n-1]}8n\left(v_{3}+v_{4}+4n\right)\right] & \\ & \;\;\;+\sum_{i\in [n]}\left(4m+4\right)\left(v_{5}+v_{6}+2m+2\right)+\sum_{i\in [n-1]}\left(4m+8\right)\left(v_{7}+v_{8}+2m+4\right)+2\sum_{i\in [n-1]}4\left(v_{9}+v_{10}\right) & \\ & \;\;\;+\sum_{i\in [2m+1]}4n\left(v_{11}+v_{12}\right)+4\left[\sum_{i\in [n]}\left(8i-4\right)\left(v_{13}+v_{14}\right)+\sum_{i\in [n-1]}8i\left(v_{15}+v_{16}\right)+\sum_{i\in [n-1]}\left[\left(8i+2\right)\left(v_{17}+v_{18}\right)\right]\right] & \\ & \;\;\;+4\left(8n-2\right)\left(v_{19}+v_{20}\right). & \end{array}$$

$$\begin{array}{ccc} S(G) & =2[2\sum_{i\in [n-1]}\left[96i^{2}+8u_{1}+16iu_{1}+16iu_{2}+8iv_{1}+8iv_{2}+80i+8u_{2}+4v_{1}+4v_{2}+4u_{1}v_{2}+4u_{2}v_{1}+16\right] & \\ & \;\;\;+\sum_{i\in [m-n-1]}\left[96n^{2}+16nu_{3}+16nu_{4}+8nv_{3}+8nv_{4}+4u_{3}v_{4}-16n+4u_{4}v_{3}\right]] & \\ & \;\;\;+\sum_{i\in [n]}\left[24m^{2}+8mu_{5}+8mu_{6}+4mv_{5}+4mv_{6}+40m+8u_{5}+8u_{6}+4v_{5}+4v_{6}+4u_{5}v_{6}+4u_{6}v_{5}+16\right] & \\ & \;\;\;+\sum_{i\in [n-1]}\left[24m^{2}+8mu_{7}+8mu_{8}+4mv_{7}+4mv_{8}+88m+16u_{7}+16u_{8}+8v_{7}+8v_{8}+4u_{7}v_{8}+4u_{8}v_{7}+80\right] & \\ & \;\;\;+2\sum_{i\in [n-1]}\left[u_{9}\left(2v_{10}+4\right)+u_{10}\left(2v_{9}+4\right)\right]+\sum_{i\in [2m+1]}\left[u_{11}\left(2v_{12}+4n\right)+u_{12}\left(2v_{11}+4n\right)\right] & \\ & \;\;\;+4[\sum_{i\in [n]}\left[u_{13}\left(2v_{14}+8i-4\right)+u_{14}\left(2v_{13}+8i-4\right)\right]+\sum_{i\in [n-1]}\left[u_{15}\left(2v_{16}+8i\right)+u_{16}\left(2v_{15}+8i\right)\right] & \\ & \;\;\;+\sum_{i\in [n-1]}\left[u_{17}\left(2v_{18}+8i+2\right)+u_{18}\left(2v_{17}+8i+2\right)\right]]+4\left[u_{19}\left(2v_{20}+8n-2\right)+u_{20}\left(2v_{19}+8n-2\right)\right]. & \end{array}$$

$$\begin{array}{ccc} Gut(G) & =2[2\sum_{i\in [n-1]}\left[2\left(4i+2\right)\left(2\left(v_{1}+v_{2}\right)+2\left(4i+2\right)\right)+2\left[\left(2v_{1}+4i+2\right)\left(2v_{2}+4i+2\right)\right]+4\left(4i+2\right)\left(4i+1\right)\right] & \\ & \;\;\;+\sum_{i\in [m-n-1]}\left[8n\left(2\left(v_{3}+v_{4}\right)+8n\right)+2\left[\left(2v_{3}+4n\right)\left(2v_{4}+4n\right)\right]+16n\left(4n-1\right)\right]] & \\ & \;\;\;+\sum_{i\in [n]}\left[2\left(2m+2\right)\left(2\left(v_{5}+v_{6}\right)+2\left(2m+2\right)\right)+2\left[\left(2v_{5}+2m+2\right)\left(2v_{6}+2m+2\right)\right]+4\left(2m+2\right)\left(2m+1\right)\right] & \\ & \;\;\;+\sum_{i\in [n-1]}\left[2\left(2m+4\right)\left(2\left(v_{7}+v_{8}\right)+2\left(2m+4\right)\right)+2\left[\left(2v_{7}+2m+4\right)\left(2v_{8}+2m+4\right)\right]+4\left(2m+4\right)\left(2m+3\right)\right] & \\ & \;\;\;+2\sum_{i\in [n-1]}\left[\left(2v_{9}+4\right)\left(2v_{10}+4\right)\right]+\sum_{i\in [2m+1]}\left[\left(2v_{11}+4n\right)\left(2v_{12}+4n\right)\right]+4[\sum_{i\in [n]}\left[\left(2v_{13}+8i-4\right)\left(2v_{14}+8i-4\right)\right] & \\ & \;\;\;+\sum_{i\in [n-1]}\left[\left(2v_{15}+8i\right)\left(2v_{16}+8i\right)\right]+\sum_{i\in [n-1]}\left[\left(2v_{17}+8i+2\right)\left(2v_{18}+8i+2\right)\right]]+4\left[\left(2v_{19}+8n-2\right)\left(2v_{20}+8n-2\right)\right]. & \end{array}$$

By substituting Table 2’s values, the proof is complete by just simplifying it. □

**Theorem** **4.** *Let G be a RK(m,n) Type-II rectangular kekulene system with m>3n−1.*

(i) *Wiener*
  - *$W\left(G\right)=\left(25920m^{3}n^{2}-2880m^{3}n+80m^{3}+8480m^{2}n^{3}+122220m^{2}n^{2}-103580m^{2}n+10140m^{2}+35640mn^{4}+18140mn^{3}+142830mn^{2}-203450mn+53245m-18792n^{5}+54720n^{4}-57320n^{3}+54675n^{2}-66748n+29490\right)/15$.*
(ii) *Edge-Wiener*
  -
$W_{e}\left(G\right)=\left(46080m^{3}n^{2}-7680m^{3}n+320m^{3}+23040m^{2}n^{3}+161280m^{2}n^{2}-153240m^{2}n+11220m^{2}+63360mn^{4}+40320mn^{3}+123360mn^{2}-239100mn+72490m-33408n^{5}+98400n^{4}-101320n^{3}+31140n^{2}-22382n+17580\right)/15.$

(iii) *Vertex-Edge-Wiener*
  -
$W_{ve}\left(G\right)=\left(43200m^{3}n^{2}-6000m^{3}n+200m^{3}+15600m^{2}n^{3}+169200m^{2}n^{2}-146580m^{2}n+8880m^{2}-69120mn^{4}+24520mn^{3}+247500mn^{2}-273700mn+69880m+91584n^{5}-59460n^{4}-95020n^{3}+157260n^{2}-115544n+35400\right)/15.$

(iv) *Vertex-Szeged*
  -
$Sz_{v}\left(G\right)=\left(146560m^{3}n^{3}+14040m^{3}n^{2}-39480m^{3}n+11760m^{3}+613080m^{2}n^{3}-372120m^{2}n^{2}-111600m^{2}n+45000m^{2}-17280mn^{4}+884080mn^{3}-983280mn^{2}+193300mn+32460m+6624n^{5}-57280n^{4}+407620n^{3}-627680n^{2}+303116n-40680\right)/15.$

(v) *Edge-Szeged*
  -
$Sz_{e}\left(G\right)=\left(276480m^{3}n^{3}-55680m^{3}n^{2}-320m^{3}n-480m^{3}+1032960m^{2}n^{3}-957120m^{2}n^{2}+115680m^{2}n-10200m^{2}+30720mn^{4}+1327840mn^{3}-1934400mn^{2}+787240mn-69540m+10752n^{5}+960n^{4}+457360n^{3}-1032960n^{2}+715988n-167160\right)/15.$

(vi) *Edge-Vertex-Szeged*
  -
$Sz_{ev}\left(G\right)=\left(408000m^{3}n^{3}-46560m^{3}n^{2}-19280m^{3}n-240m^{3}+1613440m^{2}n^{3}-1312080m^{2}n^{2}+74000m^{2}n-9840m^{2}+2195040mn^{3}-2887200mn^{2}+1021280mn-85800m+16896n^{5}-68720n^{4}+888160n^{3}-1657480n^{2}+1011584n-210240\right)/15.$

(vii) *Total-Szeged*
  -
$Sz_{t}\left(G\right)=\left(1239040m^{3}n^{3}-134760m^{3}n^{2}-78360m^{3}n+10800m^{3}+4872920m^{2}n^{3}-3953400m^{2}n^{2}+152080m^{2}n+15120m^{2}+13440mn^{4}+6602000mn^{3}-8692080mn^{2}+3023100mn-208680m+51168n^{5}-193760n^{4}+2641300n^{3}-4975600n^{2}+3042272n-628320\right)/15.$

(viii) *Padmakar–Ivan*
  -
$PI\left(G\right)=\left(6912m^{2}n^{2}-1200m^{2}n+72m^{2}+17760mn^{2}-15504mn+1248*+448n^{3}+11472n^{2}-18280n+7200\right)/3.$

(ix) *Schultz*
  -
$S\left(G\right)=\left(138240m^{3}n^{2}-19200m^{3}n+640m^{3}+62400m^{2}n^{3}+570240m^{2}n^{2}-476880m^{2}n+28440m^{2}+190080mn^{4}+119680mn^{3}+584880mn^{2}-890680mn+224240m-100224n^{5}+293520n^{4}-298720n^{3}+212160n^{2}-245216n+115680\right)/15.$

(x) *Gutman*
  -
$Gut\left(G\right)=\left(184320m^{3}n^{2}-30720m^{3}n+1280m^{3}+92160m^{2}n^{3}+714240m^{2}n^{2}-624720m^{2}n+45480m^{2}+253440mn^{4}+161280mn^{3}+674400mn^{2}-1110720mn+301960m-133632n^{5}+393600n^{4}-403040n^{3}+243360n^{2}-273088n+140880\right)/15.$

**Proof.**  With regard to m>3n−1, all of these $\Theta^{*}$-classes are as in the m=3n−1 case. We also have a few other classes as follows: $\left\{AC_{4i}:i\in \left[m-3n+1\right]\right\}$ and $\left\{OB_{4i}:i\in \left[m-3n+1\right]\right\}$. The SWQG’s vertex values of $AC_{4i}$ are $u_{21}=54n^{2}+36ni-14n-2i-16$, $u_{22}=\left|V\right|-u_{21}$, $v_{21}=72n^{2}+48ni-26n-4i-20$, and $v_{22}=\left|E\right|-v_{21}-8n$ and its edge strength is 8n. Additionally, it is simple to see the isomorphism between SWQGs of $AC_{4i}$ and $OB_{4i}$.

By applying Theorem 3’s justifications and substituting Table 2’s values, the proof is complete by just simplifying it. □

## 4. Conclusions

**Figure 13:**
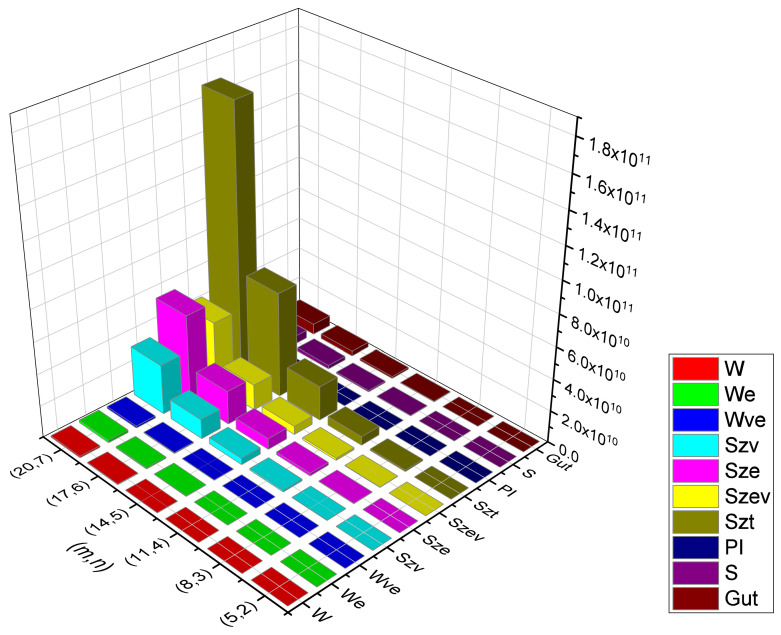
Graphical representation of RK(m,n) Type-I, m=3n−1.

**Figure 14:**
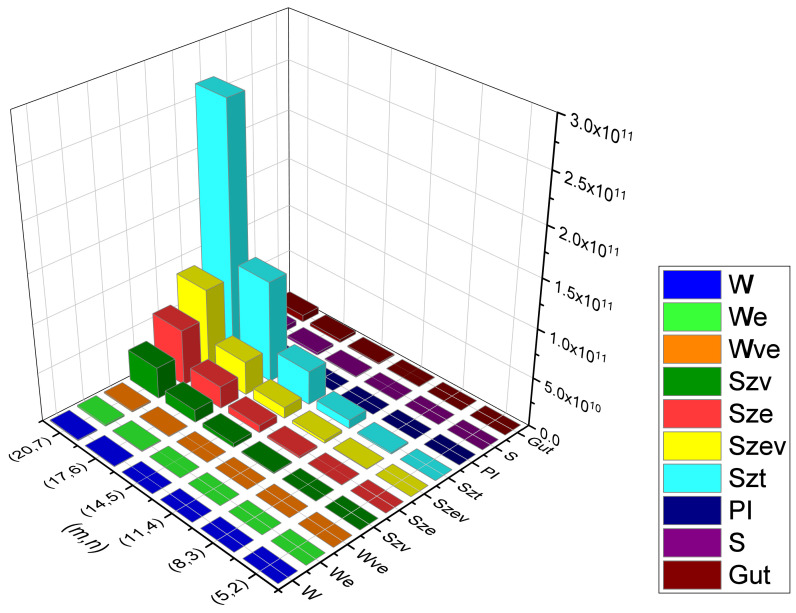
Graphical representation of RK(m,n) Type-II, m=3n−1.

**Figure 15:**
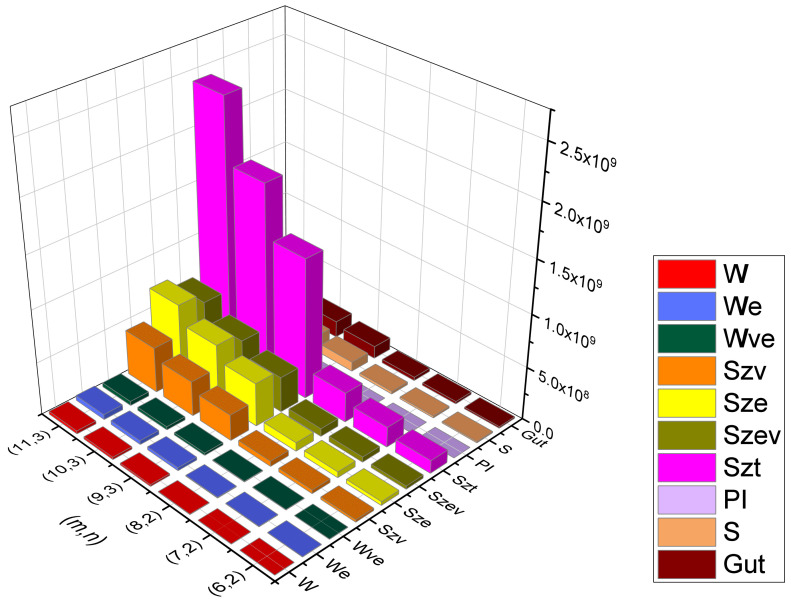
Graphical representation of RK(m,n) Type-I, m>3n−1.

**Figure 16:**
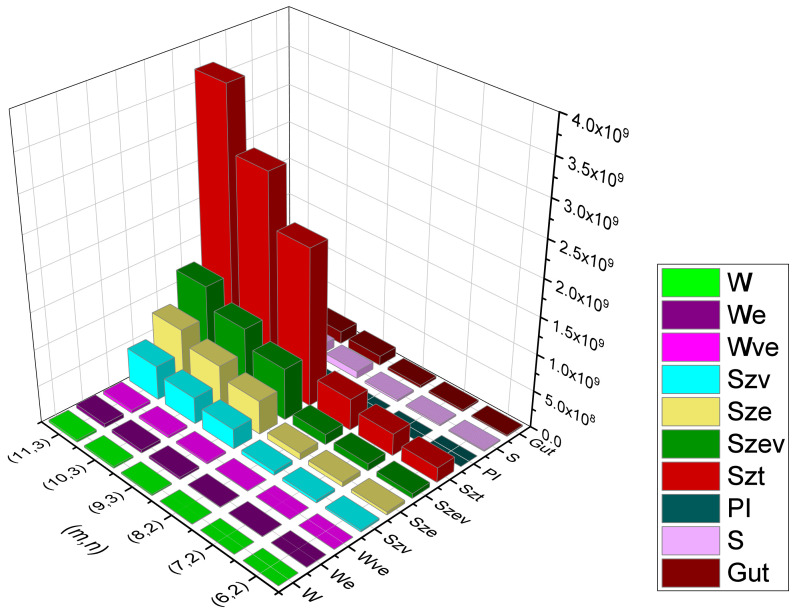
Graphical representation of RK(m,n) Type-II, m>3n−1.

Rectangular kekulene system Types (I) and (II) are the unique polycyclic aromatic compounds that we presented. We examined their topological behavioral patterns using a wide variety of molecular descriptors, i.e., we calculated the precise mathematical formulas for Wiener, Edge-Wiener, Vertex-Edge-Wiener, Vertex-Szeged, Edge-Szeged, Edge-Vertex-Szeged, Total-Szeged, Padmakar–Ivan, Schultz, and Gutman, the graphical representation of which can be seen in Figure 13, Figure 14, Figure 15 and Figure 16, numerical data can be seen in Table 3, Table 4, Table 5 and Table 6, We can easily compare the variations in each of the ten topological indices for different values of *m* and *n* from this graphical depiction. We are given a comparison between the previously defined rectangular kekulene in [53] and the rectangular kekulene Types I and II in Table 7. As the topological connectivity characteristics of these compounds are described by the molecular descriptors, the findings made in this study may be a crucial tool for comprehending the relevance of these large-sized aromatic molecules when coupled with quantum chemical descriptors in several areas, such as predictive toxicology, drug discovery, materials science, and so forth. The strength-weighted graph technique is used to calculate the analytical expressions of these tessellations. The results serve as a crucial tool for comprehending the relevance of these huge aromatic compounds in a variety of domains, including materials science and astrochemistry, as the molecular descriptors define the structural properties of the compound. Additionally, we anticipate that the analyzed structural characterization will aid in the investigation of the intriguing characteristics of these compounds, assisting in the development of novel materials with the necessary features.

## Figures and Tables

**Table 1:** Type-I rectangular kekulene quotient graphs’ strength-weighted values, m=3n−1.

QG	$w_{v}$	$s_{v}$
$G/ACZ_{1i}$ i∈[n]	$u_{1}=18i^{2}+4i$ $u_{2}=\left|V\right|-u_{1}-4i$	$v_{1}=24i^{2}+2i$ $v_{2}=\left|E\right|-v_{1}-8i$
$G/ACZ_{2i}$ i∈[m−n]	$u_{3}=18n^{2}+4n+36ni-2i$ $u_{4}=\left|V\right|-u_{3}-4n$	$v_{3}=24n^{2}+2n+48ni-4i$ $v_{4}=\left|E\right|-v_{3}-8n$
$G/ACZ_{3i}$ i∈[n−1]	$u_{5}=18i^{2}+22i-2$ $u_{6}=\left|V\right|-u_{5}-\left(4i+2\right)$	$v_{5}=24i^{2}+26i-4$ $v_{6}=\left|E\right|-v_{5}-\left(8i+4\right)$
$G/VRZ_{i}$ i∈[2n−1]	$u_{7}=18mi+16i-2m-10$ $u_{8}=\left|V\right|-u_{7}-2\left(m+1\right)$	$v_{7}=24mi+20i-4m-14$ $v_{8}=\left|E\right|-v_{7}-4\left(m+1\right)$
$G/TH_{1i}$ i∈[n]	$u_{9}=7$ $u_{10}=\left|V\right|-u_{9}$	$v_{9}=6$ $v_{10}=\left|E\right|-v_{9}-4$
$G/TH_{2i}$ i∈[n−1]	$u_{11}=7$ $u_{12}=\left|V\right|-u_{11}$	$v_{11}=6$ $v_{12}=\left|E\right|-v_{11}-4$
$G/H_{i}$ i∈[2m]	$u_{13}=18ni-i+7n$ $u_{14}=\left|V\right|-u_{13}$	$v_{13}=24ni+6n-2i$ $v_{14}=\left|E\right|-v_{13}-4n$
$G/AC_{1i}$ i∈[n]	$u_{15}=54i^{2}-68i+21$ $u_{16}=\left|V\right|-u_{15}$	$v_{15}=72i^{2}-98i+32$ $v_{16}=\left|E\right|-v_{15}-\left(8i-4\right)$
$G/AC_{2i}$ i∈[n]	$u_{17}=54i^{2}-32i+6$ $u_{18}=\left|V\right|-u_{17}$	$v_{17}=72i^{2}-50i+10$ $v_{18}=\left|E\right|-v_{17}-\left(8i-2\right)$
$G/AC_{3i}$ i∈[n−1]	$u_{19}=54i^{2}+4i$ $u_{20}=\left|V\right|-u_{19}$	$v_{19}=72i^{2}-2i$ $v_{20}=\left|E\right|-v_{19}-8i$
$G/AC_{4i}$ i∈[n]	$u_{21}=54i^{2}-50i+3$ $u_{22}=\left|V\right|-u_{21}$	$v_{21}=72i^{2}-74i+8$ $v_{22}=\left|E\right|-v_{21}-\left(8i-4\right)$
$G/AC_{5i}$ i∈[n−1]	$u_{23}=54i^{2}-14i-8$ $u_{24}=\left|V\right|-u_{23}$	$v_{23}=72i^{2}-26i-10$ $v_{24}=\left|E\right|-v_{23}-8i$
$G/AC_{6i}$ i∈[n−1]	$u_{25}=54i^{2}+22i-5$ $u_{26}=\left|V\right|-u_{25}$	$v_{25}=72i^{2}+22i-8$ $v_{26}=\left|E\right|-v_{25}-\left(8i+2\right)$
G/AC	$u_{27}=54n^{2}-14n-12$ $u_{28}=\left|V\right|-u_{27}$	$v_{27}=72n^{2}-26n-14$ $v_{28}=\left|E\right|-v_{27}-\left(8n-2\right)$

**Table 2:** Type-II rectangular kekulene quotient graphs’ strength-weighted values, m=3n−1.

QG	$w_{v}$	$s_{v}$
$G/ACZ_{1i}$ i∈[n−1]	$u_{1}=18i^{2}+22i-2$ $u_{2}=\left|V\right|-u_{1}-\left(4i+2\right)$	$v_{1}=24i^{2}+26i-4$ $v_{2}=\left|E\right|-v_{1}-2\left(4i+2\right)$
$G/ACZ_{2i}$ i∈[m−n−1]	$u_{3}=18n^{2}-14n+36ni-2i-16$ $u_{4}=\left|V\right|-u_{3}-4n$	$v_{3}=24n^{2}-22n+48ni-4i-20$ $v_{4}=\left|E\right|-v_{3}-8n$
$G/VRZ_{1i}$ i∈[n]	$u_{5}=36mi-20m+50i-44$ $u_{6}=\left|V\right|-u_{5}-2\left(m+1\right)$	$v_{5}=48mi-28m+64i-58$ $v_{6}=\left|E\right|-v_{5}-4\left(m+1\right)$
$G/VRZ_{2i}$ i∈[n−1]	$u_{7}=50mi-16m+64i-34$ $u_{8}=\left|V\right|-u_{7}-2\left(m+2\right)$	$v_{7}=48mi-4m+64i-28$ $v_{8}=\left|E\right|-v_{7}-4\left(m+2\right)$
$G/TH_{i}$ i∈[n−1]	$u_{9}=7$ $u_{10}=\left|V\right|-u_{9}$	$v_{9}=6$ $v_{10}=\left|E\right|-v_{9}-4$
$G/H_{i}$ i∈[2m+1]	$u_{11}=18ni+7n-i-17$ $u_{12}=\left|V\right|-u_{11}$	$v_{11}=24ni+6n-2i-22$ $v_{12}=\left|E\right|-v_{11}-4n$
$G/AC_{1i}$ i∈[n]	$u_{13}=54i^{2}-50i+3$ $u_{14}=\left|V\right|-u_{13}$	$v_{13}=72i^{2}-74i+8$ $v_{14}=\left|E\right|-v_{13}-\left(8i-4\right)$
$G/AC_{2i}$ i∈[n−1]	$u_{15}=54i^{2}-14i-8$ $u_{16}=\left|V\right|-u_{15}$	$v_{15}=72i^{2}-26i-10$ $v_{16}=\left|E\right|-v_{15}-8i$
$G/AC_{3i}$ i∈[n−1]	$u_{17}=54i^{2}+22i-5$ $u_{18}=\left|V\right|-u_{17}$	$v_{17}=72i^{2}+22i-8$ $v_{18}=\left|E\right|-v_{17}-\left(8i+2\right)$
G/AM	$u_{19}=54n^{2}-14n-12$ $u_{20}=\left|V\right|-u_{19}$	$v_{19}=72n^{2}-26n-14$ $v_{20}=\left|E\right|-v_{19}-\left(8n-2\right)$

**Table 3:** Numerical data for various topological indices of RK(m,n) Type-I rectangular kekulene, m=3n−1.

TI	n=2,m=5	n=3,m=8	n=4,m=11	n=5,m=14	n=6,m=17	n=7,m=20
*W*	1,529,326	12,892,583	56,651,252	176,719,457	445,629,018	971,766,315
$W_{e}$	2,441,352	21,357,446	95,542,356	301,219,250	764,945,248	1,676,476,958
$W_{ve}$	1,932,744	16,595,584	73,574,892	230,728,252	583,866,944	1,276,405,096
$Sz_{v}$	14,122,936	178,900,160	1,049,163,296	4,093,341,864	12,391,103,016	31,532,413,808
$Sz_{e}$	22,602,448	296,922,164	1,771,941,688	6,984,978,108	21,289,569,952	54,441,498,564
$Sz_{ev}$	17,870,172	230,496,884	1,363,540,172	5,347,312,020	16,242,326,140	41,433,430,836
$Sz_{t}$	72,465,728	936,816,092	5,548,185,328	21,772,944,012	66,165,325,248	168,840,774,044
PI	261,520	1,461,944	4,817,200	12,030,552	25,302,928	47,332,920
*S*	7,935,312	67,508,352	297,984,656	932,079,328	2,354,695,504	5,141,520,960
Gut	10,293,184	88,370,384	391,844,240	1,229,017,952	3,110,525,856	6,800,795,568

**Table 4:** Numerical data for various topological indices of RK(m,n) Type-II rectangular kekulene, m=3n−1.

TI	n=2,m=5	n=3,m=8	n=4,m=11	n=5,m=14	n=6,m=17	n=7,m=20
*W*	1,722,529	14,156,655	60,785,723	186,359,801	463,838,173	1,001,303,643
$W_{e}$	2,761,794	23,703,170	104,161,626	324,060,362	814,813,138	1,772,189,810
$W_{ve}$	2,181,608	18,339,132	79,752,744	246,548,292	617,258,824	1,338,352,780
$Sz_{v}$	16,210,052	198,646,488	1,132,439,628	4,327,184,312	12,897,023,156	32,430,540,184
$Sz_{e}$	26,031,212	334,230,956	1,953,733,580	7,584,990,380	22,857,516,332	57,945,771,308
$Sz_{ev}$	41,091,656	516,150,168	2,984,300,216	11,509,390,152	34,529,108,840	87,252,559,544
$Sz_{t}$	124,424,576	1,565,177,780	9,054,773,640	34,930,954,996	104,812,757,168	264,881,430,580
PI	286,504	1,579,944	5,137,720	12,704,568	26,522,888	49,332,744
*S*	8,949,992	74,572,496	322,939,352	995,869,824	2,489,185,224	5,390,821,680
Gut	11,625,280	97,990,320	426,964,400	1,321,733,792	3,312,442,336	7,187,653,584

**Table 5:** Numerical data for various topological indices of RK(m,n) Type-I rectangular kekulene, m>3n−1.

TI	n=2,m=6	n=2,m=7	n=2,m=8	n=3,m=9	n=3,m=10	n=3,m=11
*W*	2,382,825	3,509,404	4,948,263	17,307,562	22,640,917	28,982,536
$W_{e}$	3,840,674	5,699,828	8,086,526	28,793,524	37,803,722	48,544,840
$W_{ve}$	3,163,474	4,832,900	7,005,422	23,012,176	30,916,824	40,457,928
$Sz_{v}$	22,992,284	34,960,304	50,491,572	247,647,280	332,059,920	433,745,600
$Sz_{e}$	37,104,636	56,790,072	82,453,956	412,500,280	554,800,556	726,607,952
$Sz_{ev}$	31,416,480	50,319,732	75,393,800	336,115,552	469,051,260	632,132,168
$Sz_{t}$	122,929,880	192,389,840	283,733,128	1,332,378,664	1,824,962,996	2,424,617,888
PI	363,720	482,800	618,760	1,819,248	2,215,672	2,651,216
*S*	12,386,344	18,267,792	25,785,736	90,702,304	118,735,472	152,082,736
Gut	16,096,080	23,772,112	33,592,128	118,832,080	155,668,576	199,507,072

**Table 6:** Numerical data for various topological indices of RK(m,n) Type-II rectangular kekulene, m>3n−1.

TI	n=2,m=6	n=2,m=7	n=2,m=8	n=3,m=9	n=3,m=10	n=3,m=11
*W*	2,643,044	3,846,719	5,372,754	18,846,722	24,482,893	31,155,056
$W_{e}$	4,275,428	6,266,558	8,802,896	31,661,632	41,247,974	52,618,996
$W_{ve}$	3,511,224	5,296,216	7,600,984	25,190,464	33,579,148	43,653,584
$Sz_{v}$	25,849,432	38,707,996	55,250,320	271,762,600	360,917,096	467,700,696
$Sz_{e}$	41,831,848	63,024,532	90,404,464	458,729,552	610,907,812	793,550,696
$Sz_{ev}$	65,777,296	98,796,088	141,363,616	707,258,416	940,568,808	1,220,296,896
$Sz_{t}$	199,235,872	299,324,704	428,382,016	2,145,008,984	2,852,962,524	3,701,845,184
PI	393,104	516,584	656,944	1,950,656	2,360,488	2,809,440
*S*	13,754,496	20,042,792	28,020,960	99,313,648	129,050,688	164,258,496
Gut	17,894,240	26,106,992	36,534,384	130,568,400	169,736,320	216,121,280

**Table 7:** Comparison of numerical data of RK(m,n) Type-I and Type-II rectangular kekulene defined in [53].

S. No.	Des	RK(m,n)−I	RK(m,n)−II	Old RK(m,n)	RK(m,n)−I	RK(m,n)−II	Old RK(m,n)
		n=2,m=5	n=2,m=5	n=2,m=5	n=3,m=8	n=3,m=8	n=3,m=8
1	|V|	396	414	378	926	962	890
2	|E|	516	540	492	1216	1264	1168
3	*W*	1,529,326	1,722,529	1,351,715	12,892,583	14,156,655	11,606,991
4	$W_{e}$	2,441,352	2,761,794	2,147,798	21,357,446	23,703,170	19,178,010
5	$W_{ve}$	1,932,744	2,181,608	1,704,356	16,595,584	18,339,132	14,921,548
6	$Sz_{v}$	14,122,936	16,210,052	12,220,444	178,900,160	198,646,488	158,477,928
7	$Sz_{e}$	22,602,448	26,031,212	19,484,452	296,922,164	334,230,956	262,471,644
8	$Sz_{ev}$	17,870,172	4,1091,656	15,732,036	230,496,884	516,150,168	212,184,156
9	$Sz_{t}$	72,465,728	124,424,576	63,168,968	936,816,092	1,565,177,780	845,317,884
10	PI	261,520	286,504	253,032	1,461,944	1,579,944	1,445,120
11	*S*	7,935,312	8,949,992	7,003,400	67,508,352	74,572,496	60,725,712
12	Gut	10,293,184	11,625,280	907,0928	88,370,384	97,990,320	79,424,784

## Data Availability

Our study did not report any data.

## Sample Availability

Samples of the compounds are not applicable, our research does not need any samples.
